# Microstructural alterations of parafascicular nucleus pathways associated with freezing of gait in Parkinson’s disease

**DOI:** 10.1093/braincomms/fcag154

**Published:** 2026-05-20

**Authors:** Qin Niu, Shuting Bu, Xiaolu Li, Huize Pang, Yu Liu, Mengwan Zhao, Juzhou Wang, Lina Huang, Le Liang, Hongmei Yu, Yueluan Jiang, Guoguang Fan

**Affiliations:** Department of Radiology, The First Hospital of China Medical University, Shenyang 110001, China; Department of Radiology, The First Hospital of China Medical University, Shenyang 110001, China; Department of Radiology, The First Hospital of China Medical University, Shenyang 110001, China; Department of Radiology, The First Hospital of China Medical University, Shenyang 110001, China; Department of Radiology, The First Hospital of China Medical University, Shenyang 110001, China; Department of Radiology, The First Hospital of China Medical University, Shenyang 110001, China; Department of Radiology, The First Hospital of China Medical University, Shenyang 110001, China; Department of Radiology, The First Hospital of China Medical University, Shenyang 110001, China; Department of Radiology, The First Hospital of China Medical University, Shenyang 110001, China; Department of Neurology, The First Hospital of China Medical University, Shenyang 110001, China; MR Research Collaboration, Siemens Healthineers, Beijing 100102, China; Department of Radiology, The First Hospital of China Medical University, Shenyang 110001, China

**Keywords:** Parkinson’s disease, freezing of gait, diffusion tensor imaging, neurite orientation dispersion and density imaging, probabilistic tractography

## Abstract

The parafascicular nucleus (Pf) projects to the dorsal putamen (Dpu), subthalamic nucleus (STN) and nucleus accumbens (Nac), regions implicated in both motor and non-motor symptoms of Parkinson’s disease. However, microstructural alterations of these pathways across different Parkinson’s disease motor subtypes remain unclear and heterogeneity within the postural instability and gait disorder (PIGD) subtype, particularly with respect to the presence or absence of freezing of gait (FOG), has often been overlooked. Therefore, this study employed diffusion tensor imaging (DTI) and neurite orientation dispersion and density imaging (NODDI) to examine microstructural properties of Pf projections in patients with tremor-dominant Parkinson’s disease (PDTD; *n* = 25), PIGD (*n* = 34) and healthy controls (HC; *n* = 38). PIGD patients were further divided into patients with FOG (PIGD-FOG; *n* = 21) and patients without FOG (PIGD-nFOG; *n* = 13), and microstructural metrics were comprehensively assessed and compared between the two subgroups. The discrimination effects of microstructural metrics in Pf pathways were detected using receiver operating characteristic (ROC) analyses, and the correlations between microstructural metrics and clinical features of Parkinson’s disease were further analysed. Group comparisons revealed significant differences between the PIGD and HC groups, whereas no significant differences were observed between the PDTD and HC groups or between the PDTD and PIGD groups. Compared with HC, PIGD patients exhibited significant microstructural impairments in Pf-related pathways, with reduced fractional anisotropy (FA) and neurite density (NDI), along with increased axial diffusivity (AD), mean diffusivity (MD) and radial diffusivity (RD) (*P* < 0.05). Within the PIGD group, patients with FOG showed more severe degeneration of Pf pathways, particularly in the bilateral Pf-STN pathways. Specifically, FA and NDI values were reduced, while MD and RD values were increased in PIGD-FOG patients compared with PIGD-nFOG patients (*P* < 0.05). ROC analyses demonstrated that microstructural metrics in Pf pathways effectively discriminated PIGD-FOG from PIGD-nFOG patients. Moreover, microstructural impairments of Pf pathways were associated with more severe gait dysfunction, more advanced disease stage and poorer motor-related activities of daily living in PIGD patients and with greater FOG severity, cognitive impairment and worse motor-related activities of daily living in the PIGD-FOG subgroup. These findings indicate that microstructural alterations of Pf pathways are preferentially associated with the PIGD subtype and are more pronounced in patients with FOG. Together, these results underscore a strong association between Pf-related circuits and FOG in PIGD and highlight the importance of symptom-driven heterogeneity within motor subtypes.

## Teaser

Niu *et al*. reported that microstructural alterations of parafascicular nucleus pathways were associated with the postural instability and gait disorder subtype and were more pronounced in patients with freezing of gait, underscoring a strong association with freezing of gait. These results highlight the importance of symptom-driven heterogeneity within motor subtypes.

## Graphical Abstract

**Graphical Abstract:**
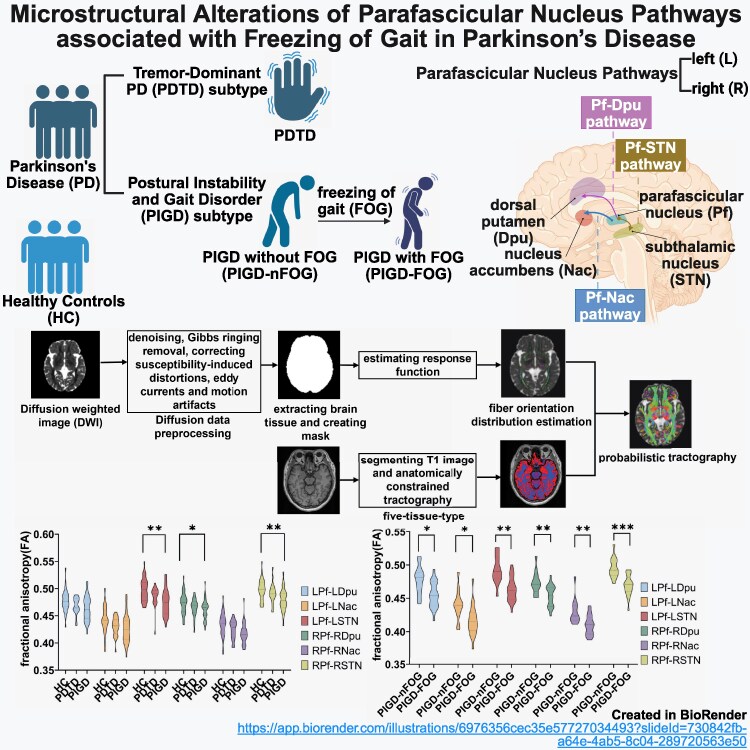

## Introduction

Parkinson’s disease is a progressive neurodegenerative disorder characterized by rest tremor, bradykinesia, rigidity and postural instability.^1^ Parkinson’s disease is usually divided into tremor-dominant (PDTD) and postural instability and gait disorder (PIGD) subtypes.^2^ The clinical course and prognosis of Parkinson’s disease vary among different motor subtypes. PDTD patients feature predominant tremor symptoms and a more benign course, whereas PIGD patients present axial motor impairments with postural instability and falls and freezing of gait (FOG) and generally exhibit more rapid progression of motor, non-motor and cognitive symptoms.^3,4^ Among these axial manifestations, FOG is a common motor impairment that gradually emerges as the disease progresses, increasing the risk of falls and impacting mobility and quality of life. Although particularly common in severe cases, especially the PIGD subtype, FOG does not manifest in all affected individuals. The pathological mechanisms underlying this debilitating symptom, however, remain unclear.

The parafascicular nucleus (Pf) is an important part of the thalamic intralaminar nucleus complex, which is closely linked to the basal ganglia (BG) and significantly contributes to motor function.^5,6^ Pf receives primary inputs from the somatosensory cortex, superior colliculus, vestibular nucleus, motor cortex and substantia nigra reticulata (SNr) and provides primary projections to the BG.^7^ Experiments based on animal models revealed that the Pf projected to three brain regions, namely, the dorsal putamen (Dpu), the subthalamic nucleus (STN) and the nucleus accumbens (Nac),^8^ and all of which were implicated in the motor and non-motor symptoms of Parkinson’s disease. However, only one patient-based study has been conducted to date, which employed spectral dynamic causal modelling to demonstrate that Pf-related projections were impaired and associated with clinical symptoms in Parkinson’s disease patients.^9^ Notably, the investigation was not conducted at the level of Parkinson’s disease motor subtypes. Therefore, further studies are needed to investigate microstructural alterations of these pathways across different Parkinson’s disease motor subtypes.

Diffusion tensor imaging (DTI) assesses the diffusion characteristics of water molecules, which can be modelled as a tensor to derive scalar metrics such as fractional anisotropy (FA), axial diffusivity (AD), radial diffusivity (RD) and mean diffusivity (MD).^10^ Several studies have shown that different Parkinson’s disease motor subtypes exhibit distinct microstructural changes in brain regions.^11-13^ Neurite Orientation Dispersion and Density Imaging (NODDI) can estimate the microstructure of the dendrites and axons by quantifying neurite density (NDI), orientation dispersion (ODI) and the free water fraction (FWF).^14,15^ Existing NODDI studies have mainly focused on specific brain regions or neural circuits in Parkinson’s disease, while correlation studies investigating differences among motor subtypes are still insufficient.^16,17^ Combining these imaging metrics provides a more detailed understanding of the microstructural alterations in Parkinson’s disease patients. However, previous neuroimaging studies comparing the PIGD and PDTD subtypes have produced inconsistent conclusions.^11,13,18,19^ This inconsistency may be attributed to the considerable heterogeneity within the PIGD subtype, particularly regarding the presence or absence of FOG. This key internal variation has often been overlooked in prior studies, which have primarily compared PIGD and PDTD subtypes without further stratifying PIGD patients based on FOG status. Moreover, deep brain stimulation (DBS) targeting the Pf has been shown to improve FOG symptoms.^20^ Despite this clinical benefit, the microstructural changes of Pf projections underlying FOG in Parkinson’s disease patients remain poorly understood.

Therefore, our study used DTI and NODDI to explore the microstructural alterations of Pf projections in Parkinson’s disease motor subtypes, with a specific focus on comparing PIGD patients with and without FOG. We further assessed the discriminative performance of these microstructural metrics in Pf pathways for PIGD-FOG. In addition, we explored the associations between microstructural alterations of Pf projections and clinical measures in Parkinson’s disease. We hypothesized the following key findings: (i) The PIGD subtype exhibited microstructural degeneration of Pf pathways. (ii) Within the PIGD subtype, patients with FOG exhibited more severe degeneration of Pf pathways compared to those without FOG. (iii) The microstructural metrics in Pf pathways demonstrated strong discriminative performance in distinguishing PIGD-FOG from PIGD-nFOG.

## Materials and methods

### Participants

Between September 2023 and April 2025, a total of 59 individuals diagnosed with Parkinson’s disease (34 males, 25 females) together with 38 HC (13 males, 25 females) were recruited from the Department of Neurology at the First Hospital of China Medical University. The diagnosis of Parkinson’s disease was established according to the UK Brain Bank criteria.^21^ Classification of motor subtypes was based on the ratio of the mean tremor score (11 items) to the mean gait score (5 items) as assessed by the Movement Disorder Society Unified Parkinson’s Disease Rating Scale (MDS-UPDRS). Patients with a ratio ≥ 1.15 were designated as PDTD subtype, those with a ratio ≤ 0.9 as PIGD subtype, and those with ratios between 0.9 and 1.15 as intermediate subtype.^22^ Then in the 34 PIGD patients, 21 and 13 patients had been diagnosed with and without FOG, respectively, according to the Freezing of Gait Questionnaire (FOGQ) and MDS-UPDRS Part III 3.11 item. No cases of FOG were observed in the PDTD group. Participants meeting the inclusion criteria were aged between 40 and 80 years, right-handed and of Han ethnicity. Individuals were excluded if they had a history of neurological surgery, psychological diseases and any contraindication to MRI or if they were classified as the intermediate subtype. To minimize the influence of medication, participants were required to take their medication at least 12 h prior to the clinical and MRI examinations. Demographic information, such as age, sex and years of education, was collected for all participants prior to MRI acquisition. For the Parkinson’s disease patients, additional data on disease duration and levodopa equivalent daily dose (LEDD) were recorded. All participants underwent comprehensive clinical assessments. Cognitive function was evaluated using the Mini-Mental State Examination (MMSE) and the Montreal Cognitive Assessment (MOCA). Symptom severity in Parkinson’s disease patients was quantified using the MDS-UPDRS Part III and the Hoehn and Yahr (H&Y) scale, while FOG severity was quantified via the FOGQ. Furthermore, anxiety and depressive symptoms in Parkinson’s disease patients were evaluated using the Hamilton Anxiety Scale (HAMA) and the Hamilton Depression Scale (HAMD). Prior to participation, all participants provided written informed consent, and the study protocol received approval from the Institutional Review Board of China Medical University (IRB No. 2020-52).

### MRI acquisition

All participants underwent MRI scanning on a 3.0 T MRI scanner (MAGNETOM Vida, Siemens Healthcare, Erlangen, Germany) equipped with a 64-channel phased-array head coil. Two-shell high-angular resolution diffusion imaging (HARDI) data were acquired for each participant. The primary acquisition parameters were as follows: field of view (FOV) = 220 × 220 mm^2^, matrix size = 110 × 110, 129 slices, voxel size = 2 × 2 × 2 mm^3^, repetition time (TR) = 5500 ms, echo time (TE) = 85 ms and flip angle = 90°. The diffusion-weighted imaging (DWI) data included 1 non-DW image (*b* = 0 s/mm^2^, B0) with posterior–anterior (PA) phase encoding, 1 non-DW image (*b* = 0 s/mm^2^, B0) with anterior–posterior (AP) phase encoding and 128 DW images acquired along 128 non-collinear gradient directions (64 directions at *b* = 1000 s/mm^2^ and 64 directions at *b* = 2000s/mm^2^) with AP phase encoding.

In addition, a three-dimensional (3D) T1-weighted image was obtained for each participant using a magnetization prepared rapid gradient echo (MPRAGE) sequence. Imaging parameters were as follows: FOV = 260 × 260 mm^2^, matrix size = 256 × 256, 176 sagittal slices, voxel size = 1 × 1 × 1 mm^3^, TR = 2000ms, TE = 1.91 ms and flip angle = 9°. All scans were conducted at least 12 h after the last dose of anti-Parkinson’s medication.

### Diffusion data preprocessing

**Figure 1:**
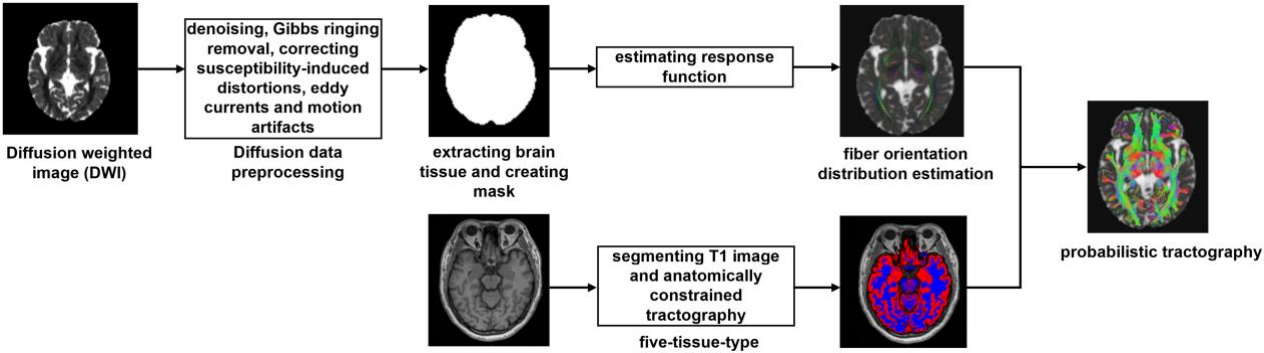
Processing pipeline of probabilistic tractography preparation.

Diffusion data were processed with MRtrix Version 3.0.3 and FSL Version 6.0.6.4. Raw diffusion images were initially denoised and corrected for Gibbs ringing artefacts in MRtrix,^23-26^ followed by corrections for susceptibility-induced distortions, eddy currents and head motion using FSL.^27-29^ Field inhomogeneities were corrected with the N4 bias field correction algorithm implemented in Advanced Normalization Tools (ANTs).^30^ The preprocessed DWI images were then co-registered to the T1 space using FSL-FLIRT.^31,32^ A five-tissue-type segmented T1 image was used to generate a tissue segmentation image and a mask suitable for seeding streamlines at the grey matter-white matter (WM) interface.^33^ Probabilistic tractography was performed in MRtrix using fibre orientation distribution (FOD) estimated through constrained spherical deconvolution (CSD). The procedural pipelines for the preparation of probabilistic tractography are shown in Fig. 1. Diffusion tensor estimation was conducted using a weighted least-squares approach,^34,35^ and the response function for CSD was estimated accordingly.^36^

### Definition of regions of interest

The Pf was identified using a histologically derived probabilistic atlas.^37^ Segmentation of thalamic nuclei was conducted with FreeSurfer (http://surfer.nmr.mgh.harvard.edu), utilizing an integrated probabilistic thalamic segmentation algorithm. Preprocessing of T1-weighted images included the ‘recon-all’ pipeline, which performed motion correction, intensity normalization, skull stripping and automated segmentation of neuroanatomical structures. Subsequent Bayesian inference analysis partitioned each thalamus into 25 distinct nuclei based on the aforementioned probabilistic atlas. The left and right Pf were then extracted as individual masks for each participant. The location of the Dpu and Nac was based on the human Brainnetome atlas (BN),^38^ while the STN was based on the atlas of the basal ganglia (ATAG).^39^

### Probabilistic tractography

Tractography was performed following these settings: minimum fibre length = 20 mm, maximum fibre length = 100 mm, maximum angle = 45°, minimal fibre orientation distribution function (fODF) amplitude = 0.05. The fibre tractography results are presented in Supplementary Fig. 1.

### NODDI

The Accelerated Microstructure Imaging via Convex Optimization (AMICO) package (https://github.com/daducci/AMICO) NODDI model was applied to preprocessed data.^40^

### Statistical analysis

All statistical analyses were conducted using the IBM SPSS Statistics software (Version 25). The Shapiro–Wilk method test was employed to assess the normal distribution of demographic and clinical data. Normally distributed measurement data were analysed using one-way analysis of variance (ANOVA), followed by Bonferroni-corrected *post hoc t*-tests with a significance threshold of *P* < 0.05. Data that were not normally distributed or showed unequal variances were assessed using the corresponding rank-sum tests, either the Mann–Whitney *U* or Kruskal–Wallis H test. Categorical variables were compared using the chi-square test. Additionally, group comparisons of DTI and NODDI metrics were conducted using general linear models (GLMs). A separate univariate GLM was conducted for each tract-metric combination to assess microstructural alterations in Pf-related pathways. Initially, GLMs were used to compare microstructural metrics among HC and Parkinson’s disease motor subtypes, with age, sex and years of education included as covariates. *Post hoc* pairwise comparisons among the groups were conducted within each GLM and corrected for multiple comparisons using the Bonferroni correction. Subsequently, PIGD participants were further classified into PIGD-FOG and PIGD-nFOG groups, with age, disease duration and MDS-UPDRS Part III scores included as covariates. Fisher’s exact test was used for categorical variables in the comparison between the PIGD-FOG and PIGD-nFOG groups. In addition, to assess whether motor symptom laterality influenced the main findings, additional GLM analyses were performed with laterality included as an additional covariate. The criteria used to classify motor symptom laterality were described in detail in the Supplementary material. Effect sizes were reported as partial eta squared (*η*^2^), and statistical significance was set at *P* < 0.05 after correction. Receiver operating characteristic (ROC) curves were utilized to assess the discriminative performance of the microstructural metrics in distinguishing between different groups. For each Pf-related pathway, an independent ROC model was constructed by including all microstructural metrics that showed significant intergroup differences in either hemisphere within that pathway. Statistical comparisons of the area under the curve (AUC) values among pairs of models were performed using DeLong’s test. The threshold for statistical significance was set at *P* < 0.05.

### Clinical correlations

To examine the associations between microstructural alterations of Pf projections and clinical features, partial correlation analyses were performed between imaging metrics and clinical measures. For Parkinson’s disease motor subtypes, age, sex and years of education were included as covariates, while for the PIGD subgroup, age, disease duration and MDS-UPDRS Part III scores were included as covariates.

## Results

### Demographic and clinical information

The demographic and clinical features of the participants are shown in Tables 1 and 2. No significant differences were found in sex, years of education or MMSE scores among the Parkinson’s disease motor subtypes and HC (*P* > 0.05). However, both Parkinson’s disease motor subtypes were significantly older and exhibited lower MOCA scores compared with the HC group (*P* < 0.05). Significant differences were observed between the PIGD and PDTD subtypes in gait and tremor scores, MDS-UPDRS Part II and III scores, H&Y stage, disease duration and LEDD (*P* < 0.05). No significant differences in HAMA and HAMD scores were observed between the two Parkinson’s disease motor subtypes (*P* > 0.05). Within the PIGD subtype, no significant differences were found between the PIGD-FOG and PIGD-nFOG groups in sex, age, years of education, disease duration, LEDD, tremor scores, MOCA, MMSE, HAMA or HAMD scores (*P* > 0.05). In contrast, compared with PIGD-nFOG patients, the PIGD-FOG group demonstrated significantly higher scores in gait performance, FOGQ and MDS-UPDRS Parts II and III, as well as more advanced H&Y stage (*P* < 0.05).

**Table 1:** Demographic and clinical information among Parkinson’s disease motor subtypes

Characteristics	HC (*n* = 38)	Parkinson’s disease (*n* = 59)		H/*Z*/*t*/*χ*^2^	*P*-value	*P*-value (*post hoc* test)		
		PDTD (*n* = 25)	PIGD (*n* = 34)			HCvsPDTD	HCvsPIGD	PDTDvsPIGD
Sex (male/female)	13/25	14/11	20/14	5.120	0.077	0.441	0.191	1.000
Age (years)	58.00 (12.50)	65.00 (10.00)	67.00 (10.25)	18.903	**<0.001** ^a,b^	**0.019**	**<0.001**	0.777
Education (years)	10.00 (3.50)	9.00 (3.00)	9.00 (4.00)	2.770	0.250	1.000	0.342	0.744
Duration (years)	NA	2.50 (3.50)	6.13 (6.50)	−2.383	**0**.**017**^c^	NA	NA	**0**.**017**
H&Y	NA	2.00 (0.50)	3.00 (2.00)	−3.548	**<0**.**001**^c^	NA	NA	**<0**.**001**
MDS-UPDRS Part II	NA	7.00 (11.50)	16.00 (12.50)	−3.756	**<0**.**001**^c^	NA	NA	**<0**.**001**
MDS-UPDRS Part III	NA	31.00 (23.00)	44.50 (18.00)	−2.340	**0**.**019**^c^	NA	NA	**0**.**019**
Gait score	NA	2.00 (1.50)	7.00 (9.00)	−5.469	**<0**.**001**^c^	NA	NA	**<0**.**001**
Tremor score	NA	9.00 (7.00)	4.00 (5.00)	−4.614	**<0**.**001**^c^	NA	NA	**<0**.**001**
LEDD (mg)	NA	300.00 (406.25)	431.25 (412.50)	−2.221	**0**.**026**^c^	NA	NA	**0**.**026**
MOCA	26.00 (3.25)	23.00 (6.00)	22.00 (7.25)	13.730	**0.001** ^a,b^	**0.033**	**0.001**	1.000
MMSE	28.00 (3.00)	28.00 (5.50)	26.00 (4.00)	4.777	0.092	1.000	0.079	0.486
HAMD	NA	8.00 (8.00)	11.00 (16.00)	−1.545	0.122	NA	NA	0.122
HAMA	NA	9.96 ± 6.30	13.06 ± 7.14	1.729	0.089	NA	NA	0.089

Continuous variables are expressed as median (interquartile range, IQR) for non-normally distributed data and as mean ± standard deviation for normally distributed data. Categorical variables are presented as the number of patients. Bold values indicate statistically significant differences between groups (*P* < 0.05).

H&Y, Hoehn and Yahr disability scale; MDS-UPDRS Part II, Movement Disorder Society Unified Parkinson’s Disease Rating Scale, Part II; MDS-UPDRS Part III, Movement Disorder Society Unified Parkinson’s Disease Rating Scale, Part III; LEDD, Levodopa Equivalent Daily Dosage; MOCA, Montreal Cognitive Assessment; MMSE, Mini-Mental State Examination; HAMD, Hamilton Depression Scale; HAMA, Hamilton Anxiety Scale; HC, healthy controls; PDTD, tremor-dominant patients; PIGD, postural instability and gait disorder patients.

^a^Significant differences between HC and PDTD, Bonferroni corrected.

^b^Significant differences between HC and PIGD, Bonferroni corrected.

^c^Significant differences between PDTD and PIGD, Bonferroni corrected.

**Table 2:** Demographic and clinical information within the PIGD subtype

Characteristics	PIGD-FOG (*n* = 21)	PIGD-nFOG (*n* = 13)	*t*/*Z*/*χ*^2^	*P*-value
Sex (male/female)	13/8	7/6	—	0.728
Age (years)	66.67 ± 7.86	65.23 ± 5.90	0.566	0.575
Education (years)	9.00 (2.00)	9.00 (4.50)	−0.186	0.852
Duration (years)	3.00 (7.34)	6.50 (4.25)	−1.049	0.294
FOGQ	13.00 (7.50)	0	−4.987	**<0.001**
H&Y	3.00 (3.00)	2.00 (1.00)	−2.258	**0**.**024**
MDS-UPDRS Part II	23.10 ± 8.49	10.92 ± 4.15	5.580	**<0**.**001**
MDS-UPDRS Part III	51.76 ± 21.56	36.85 ± 11.76	2.284	**0**.**029**
Gait score	10.00 (10.00)	3.00 (4.00)	−4.023	**<0**.**001**
Tremor score	4.00 (5.50)	3.00 (3.00)	−0.661	0.509
LEDD (mg)	337.50 (462.50)	450.00 (449.50)	−0.498	0.619
MOCA	20.76 ± 4.86	22.54 ± 4.61	−1.056	0.299
MMSE	26.00 (4.00)	27.00 (3.00)	−1.142	0.253
HAMD	13.00 (13.50)	10.00 (22.00)	−0.710	0.477
HAMA	14.67 ± 7.26	10.46 ± 6.37	1.717	0.096

Continuous variables are expressed as median (interquartile range, IQR) for non-normally distributed data and as mean ± standard deviation for normally distributed data. Categorical variables are presented as the number of patients and are analysed using Fisher’s exact test due to the small sample size. Bold values indicate statistically significant differences between groups (*P* < 0.05).

FOGQ, Freezing of Gait Questionnaire; H&Y, Hoehn and Yahr disability scale; MDS-UPDRS Part II, Movement Disorder Society Unified Parkinson’s Disease Rating Scale, Part II; MDS-UPDRS Part III, Movement Disorder Society Unified Parkinson’s Disease Rating Scale, Part III; LEDD, Levodopa Equivalent Daily Dosage; MOCA, Montreal Cognitive Assessment; MMSE, Mini-Mental State Examination; HAMD, Hamilton Depression Scale; HAMA, Hamilton Anxiety Scale; PIGD, postural instability and gait disorder patients; PIGD-FOG, PIGD patients with FOG; PIGD-nFOG, PIGD patients without FOG.

### Microstructural changes in Parkinson’s disease motor subtypes

**Figure 2:**
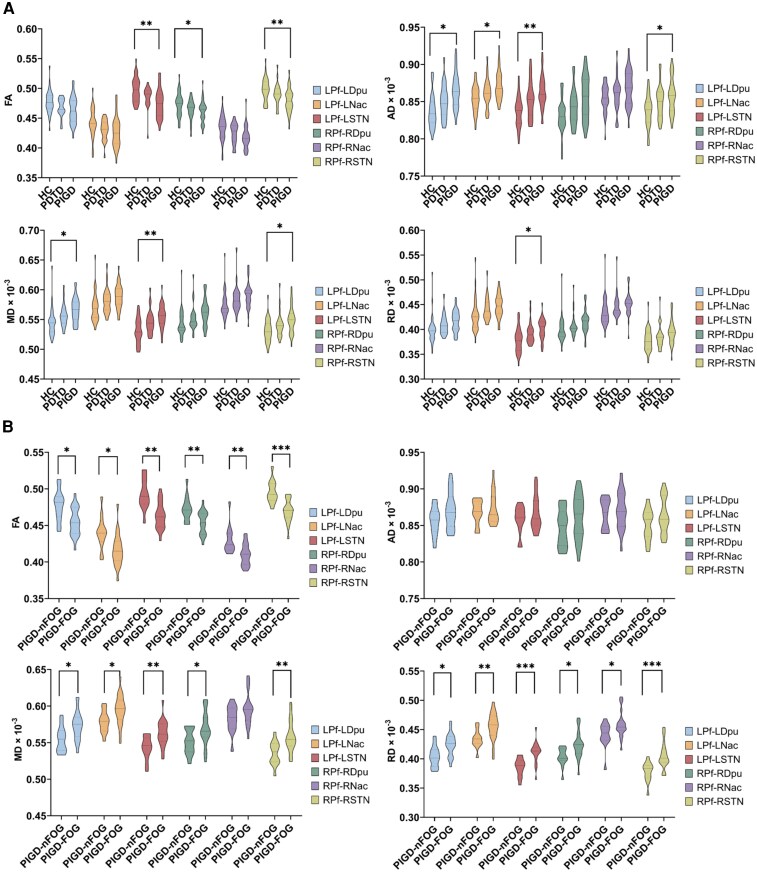
**DTI changes among groups.** (**A**) DTI changes among HC (*n* = 38) and Parkinson’s disease motor subtypes (PIGD, *n* = 34; PDTD, *n* = 25). DTI metrics in the Pf pathways are compared among Parkinson’s disease motor subtypes and HC using general linear models (GLMs), controlling for sex, age and years of education. (**B**) DTI changes in PIGD patients with and without FOG (PIGD-FOG, *n* = 21; PIGD-nFOG, *n* = 13). DTI metrics in the Pf pathways are compared between PIGD-FOG and PIGD-nFOG groups using GLMs, controlling for age, disease duration and MDS-UPDRS Part III. LPf, left parafascicular nucleus; LDpu, left dorsal putamen; LNac, left nucleus accumbens; LSTN, left subthalamic nucleus; RPf, right parafascicular nucleus; RDpu, right dorsal putamen; RNac, right nucleus accumbens; RSTN, right subthalamic nucleus; L, left; R, right; HC, healthy controls; PDTD, tremor-dominant patients; PIGD, postural instability and gait disorder patients; PIGD-FOG, PIGD patients with FOG; PIGD-nFOG, PIGD patients without FOG; FA, fractional anisotropy; AD, axial diffusivity; MD, mean diffusivity; RD, radial diffusivity. ***P < 0.001; **P < 0.01; *P < 0.05.

The results of the DTI metrics are shown in Fig. 2A, with detailed statistical comparisons among Parkinson’s disease motor subtypes and HC provided in Supplementary Table 1A. Significant *post hoc* differences were confined to comparisons between the PIGD and HC groups, whereas no significant differences were observed between the PDTD and PIGD subtypes or between the PDTD subtype and HC. Compared with HC, the PIGD group exhibited significantly reduced FA values in the bilateral Pf-STN pathways (Bonferroni-corrected: left, *P* = 0.003; right, *P* = 0.004) and the right Pf-Dpu pathway (Bonferroni-corrected, *P* = 0.032). In contrast, AD values were significantly increased in the PIGD group relative to HC in the bilateral Pf-STN pathways (Bonferroni-corrected: left, *P* = 0.007; right, *P* = 0.039), left Pf-Dpu pathway (Bonferroni-corrected, *P* = 0.015) and left Pf-Nac pathway (Bonferroni-corrected, *P* = 0.023). Similarly, the PIGD group showed significantly higher MD values in the bilateral Pf-STN pathways (Bonferroni-corrected: left, *P* = 0.005; right, *P* = 0.040) and left Pf-Dpu pathway (Bonferroni-corrected, *P* = 0.042) compared with HC. Furthermore, RD value was significantly elevated in the left Pf-STN pathway in the PIGD group compared with HC (Bonferroni-corrected, *P* = 0.019).

**Figure 3:**
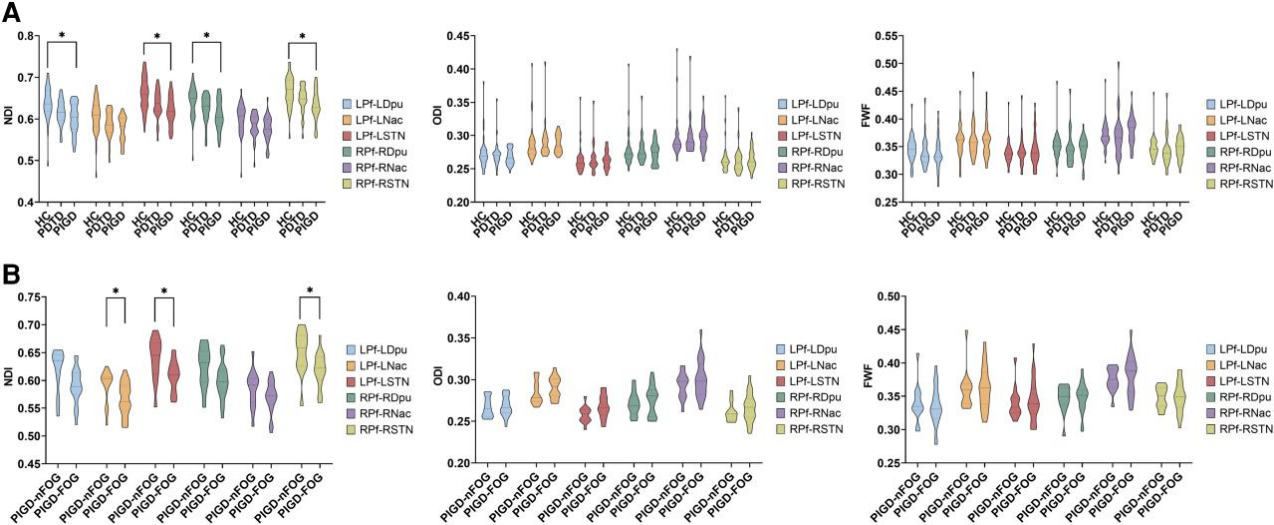
**NODDI changes among groups.** (**A**) NODDI changes among HC (*n* = 38) and Parkinson’s disease motor subtypes (PIGD, *n* = 34; PDTD, *n* = 25). NODDI metrics in the Pf pathways are compared among Parkinson’s disease motor subtypes and HC using general linear models (GLMs), controlling for sex, age and years of education. (**B**) NODDI changes in PIGD patients with and without FOG (PIGD-FOG, *n* = 21; PIGD-nFOG, *n* = 13). NODDI metrics in the Pf pathways are compared between PIGD-FOG and PIGD-nFOG groups using GLMs, controlling for age, disease duration and MDS-UPDRS Part III. LPf, left parafascicular nucleus; LDpu, left dorsal putamen; LNac, left nucleus accumbens; LSTN, left subthalamic nucleus; RPf, right parafascicular nucleus; RDpu, right dorsal putamen; RNac, right nucleus accumbens; RSTN, right subthalamic nucleus; L, left; R, right; HC, healthy controls; PDTD, tremor-dominant patients; PIGD, postural instability and gait disorder patients; PIGD-FOG, PIGD patients with FOG; PIGD-nFOG, PIGD patients without FOG; NDI, neurite density index; ODI, orientation dispersion index; FWF, free water fraction. ***P < 0.001; **P < 0.01; *P < 0.05.

*Post hoc* analyses revealed significantly reduced NDI values in the bilateral Pf-Dpu pathways (Bonferroni-corrected: left, *P* = 0.029; right, *P* = 0.030) and bilateral Pf-STN pathways (Bonferroni-corrected: left, *P* = 0.016; right, *P* = 0.027) in the PIGD group compared with HC (Fig. 3A), whereas no significant differences were observed for the PDTD group relative to either PIGD or HC. No significant group differences were detected for the remaining NODDI metrics. Detailed results are provided in Supplementary Table 1B.

### Microstructural changes in PIGD patients with and without FOG

To further investigate the microstructural alterations of Pf pathways related to FOG within the PIGD group, the PIGD subtype was stratified into PIGD-FOG and PIGD-nFOG groups, with age, disease duration and MDS-UPDRS Part III included as covariates. The PIGD-FOG group exhibited significantly more severe microstructural degeneration than the PIGD-nFOG group (Figs 2B and 3B). Compared with the PIGD-nFOG group, patients with PIGD-FOG showed significantly reduced FA values in the bilateral Pf-Dpu pathways (left, *P* = 0.022, partial *η*^2^ = 0.168; right, *P* = 0.006, partial *η*^2^ = 0.236), bilateral Pf-Nac pathways (left, *P* = 0.012, partial *η*^2^ = 0.197; right, *P* = 0.004, partial *η*^2^ = 0.255) and bilateral Pf-STN pathways (left, *P* = 0.002, partial *η*^2^ = 0.285; right, *P* < 0.001, partial *η*^2^ = 0.374). In contrast, MD values were significantly increased in the PIGD-FOG group within the bilateral Pf-Dpu pathways (left, *P* = 0.014, partial *η*^2^ = 0.190; right, *P* = 0.021, partial *η*^2^ = 0.170), bilateral Pf-STN pathways (left, *P* = 0.004, partial *η*^2^ = 0.256; right, *P* = 0.002, partial *η*^2^ = 0.275) and left Pf-Nac pathway (*P* = 0.010, partial *η*^2^ = 0.210). Similarly, RD values were significantly higher in the PIGD-FOG group in the bilateral Pf-Dpu pathways (left, *P* = 0.011, partial *η*^2^ = 0.201; right, *P* = 0.011, partial *η*^2^ = 0.201), bilateral Pf-Nac pathways (left, *P* = 0.003, partial *η*^2^ = 0.261; right, *P* = 0.035, partial *η*^2^ = 0.144) and bilateral Pf-STN pathways (left, *P* < 0.001, partial *η*^2^ = 0.339; right, *P* < 0.001, partial *η*^2^ = 0.318). Furthermore, NDI values were significantly reduced in the PIGD-FOG group in the bilateral Pf-STN pathways (left, *P* = 0.019, partial *η*^2^ = 0.175; right, *P* = 0.019, partial *η*^2^ = 0.175) and left Pf-Nac pathway (*P* = 0.039, partial *η*^2^ = 0.139). No significant differences were observed between the two groups for other microstructural metrics. Detailed results are provided in Supplementary Table 2A and B.

After additional adjustment for motor symptom laterality, significant group differences emerged in the left Pf-Dpu pathway and right Pf-STN pathway (Supplementary Table 3A and B). Specifically, AD values were increased in the left Pf-Dpu pathway (*P* = 0.044, partial *η*^2^ = 0.137) and right Pf-STN pathway (*P* = 0.042, partial *η*^2^ = 0.140), while the NDI was decreased in the left Pf-Dpu pathway (*P* = 0.043, partial *η*^2^ = 0.138) in PIGD-FOG patients compared with PIGD-nFOG patients. Additionally, microstructural metrics in Pf pathways were compared between the PIGD-nFOG and PDTD groups, with detailed results provided in Supplementary Table 4A and B.

### Discrimination between PIGD-FOG and PIGD-nFOG using ROC analyses

**Figure 4:**
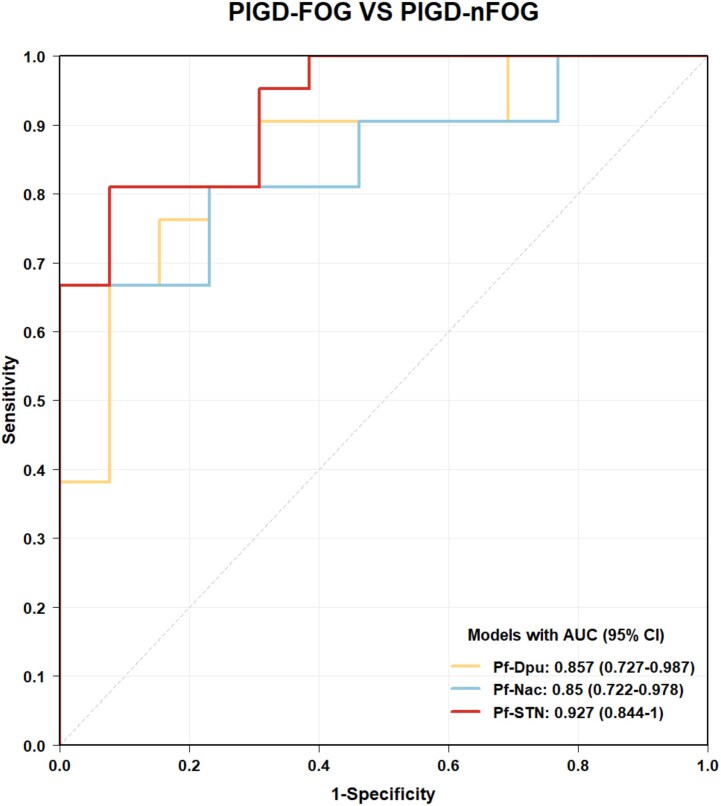
**Discriminative performance of multiple models in distinguishing PIGD-FOG (*n* = 21) from PIGD-nFOG (*n* = 13) using ROC curve analyses.** CI, confidence interval. Separate ROC curves are generated for each Pf pathway (Pf-Dpu, Pf-Nac, Pf-STN) by combining microstructural metrics showing significant intergroup differences in either hemisphere within that pathway.

For distinguishing PIGD-FOG from PIGD-nFOG, the Pf-Dpu, Pf-Nac and Pf-STN models demonstrated strong discriminative performance, with AUCs of 0.857 (95% CI 0.727–0.987), 0.850 (95% CI 0.722–0.978) and 0.927 (95% CI 0.844–1.000), respectively (Fig. 4). These results are summarized in Supplementary Table 5. Pairwise comparisons between these models revealed no significant differences (*P* > 0.05).

### Correlation analyses between imaging metrics and clinical measures

**Figure 5:**
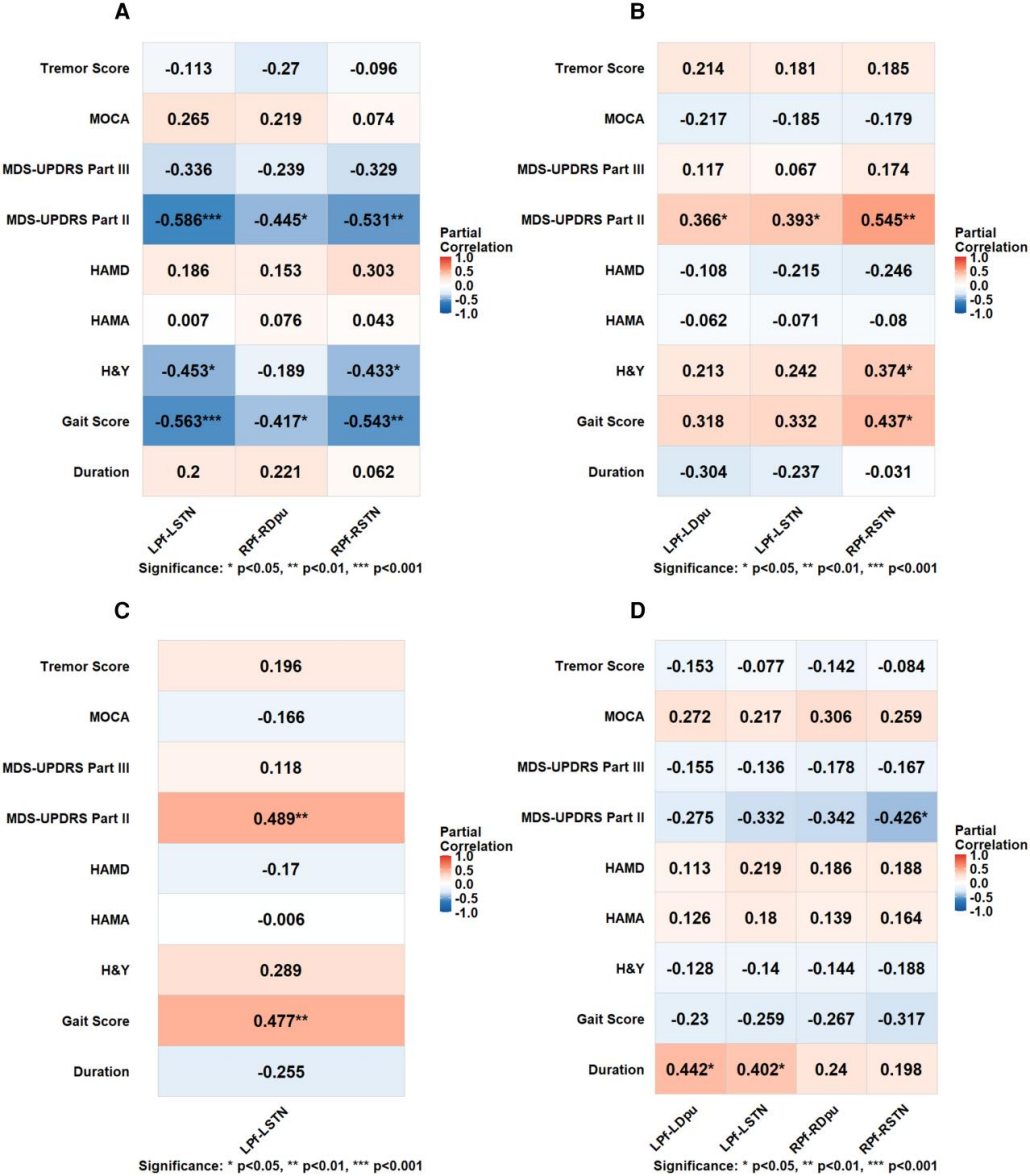
**Partial correlations between imaging metrics and clinical measures in the PIGD subtype (*n* = 34).** Partial correlations between clinical measures and FA values in Pf projections in the PIGD subtype (**A**), MD values in Pf projections in the PIGD subtype (**B**), RD values in Pf projections in the PIGD subtype (**C**) and NDI values in Pf projections in the PIGD subtype (**D**). LPf, left parafascicular nucleus; LDpu, left dorsal putamen; LNac, left nucleus accumbens; LSTN, left subthalamic nucleus; RPf, right parafascicular nucleus; RDpu, right dorsal putamen; RNac, right nucleus accumbens; RSTN, right subthalamic nucleus; L, left; R, right; H&Y, Hoehn and Yahr disability scale; MDS-UPDRS Part II, Movement Disorder Society Unified Parkinson’s Disease Rating Scale, Part II; MDS-UPDRS Part III, Movement Disorder Society Unified Parkinson’s Disease Rating Scale, Part III; MOCA, Montreal Cognitive Assessment; HAMD, Hamilton Depression Scale; HAMA, Hamilton Anxiety Scale. Significance: *P < 0.05, **P < 0.01; ***P < 0.001.

Depending on the normality of the data, partial correlation analyses (Pearson or Spearman) were conducted to evaluate the associations between neuroimaging metrics and clinical measures. Partial correlations between Pf projections and clinical measures in the PIGD subtype are shown in Fig. 5. In the PIGD subtype, MDS-UPDRS Part II and gait scores were significantly negatively correlated with FA values in the bilateral Pf-STN pathways (left, *r* = −0.586, *P* < 0.001, *r* = −0.563, *P* < 0.001, respectively; right, *r* = −0.531, *P* = 0.002, *r* = −0.543, *P* = 0.002, respectively) and right Pf-Dpu pathway (*r* = −0.445, *P* = 0.012; *r* = −0.417, *P* = 0.020, respectively). In addition, FA values in the bilateral Pf-STN pathways were negatively correlated with H&Y stage (left, *r* = −0.453, *P* = 0.011; right, *r* = −0.433, *P* = 0.015). Regarding MD measures, MDS-UPDRS Part II scores were positively correlated with MD values in the bilateral Pf-STN pathways (left, *r* = 0.393, *P* = 0.029; right, *r* = 0.545, *P* = 0.002) and left Pf-Dpu pathway (*r* = 0.366, *P* = 0.043). MD values in the right Pf-STN pathway were positively associated with both gait scores and H&Y stage (*r* = 0.437, *P* = 0.014; *r* = 0.374, *P* = 0.038, respectively). In addition, RD values in the left Pf-STN pathway were also positively correlated with MDS-UPDRS Part II and gait scores (*r* = 0.489, *P* = 0.005; *r* = 0.477, *P* = 0.007, respectively). Regarding NODDI metrics, NDI value in the right Pf-STN pathway was negatively correlated with MDS-UPDRS Part II scores (*r* = −0.426, *P* = 0.017). Positive correlations were further identified between disease duration and NDI values in the left Pf-Dpu pathway (*r* = 0.442, *P* = 0.013) and left Pf-STN pathway (*r* = 0.402, *P* = 0.025). No other diffusion metrics showed significant correlations with clinical measures in this subtype. Collectively, these findings indicated that microstructural degeneration of Pf-related projections was significantly associated with more advanced disease stage, greater impairment in motor-related activities of daily living and more severe gait dysfunction in the PIGD subtype.

**Figure 6:**
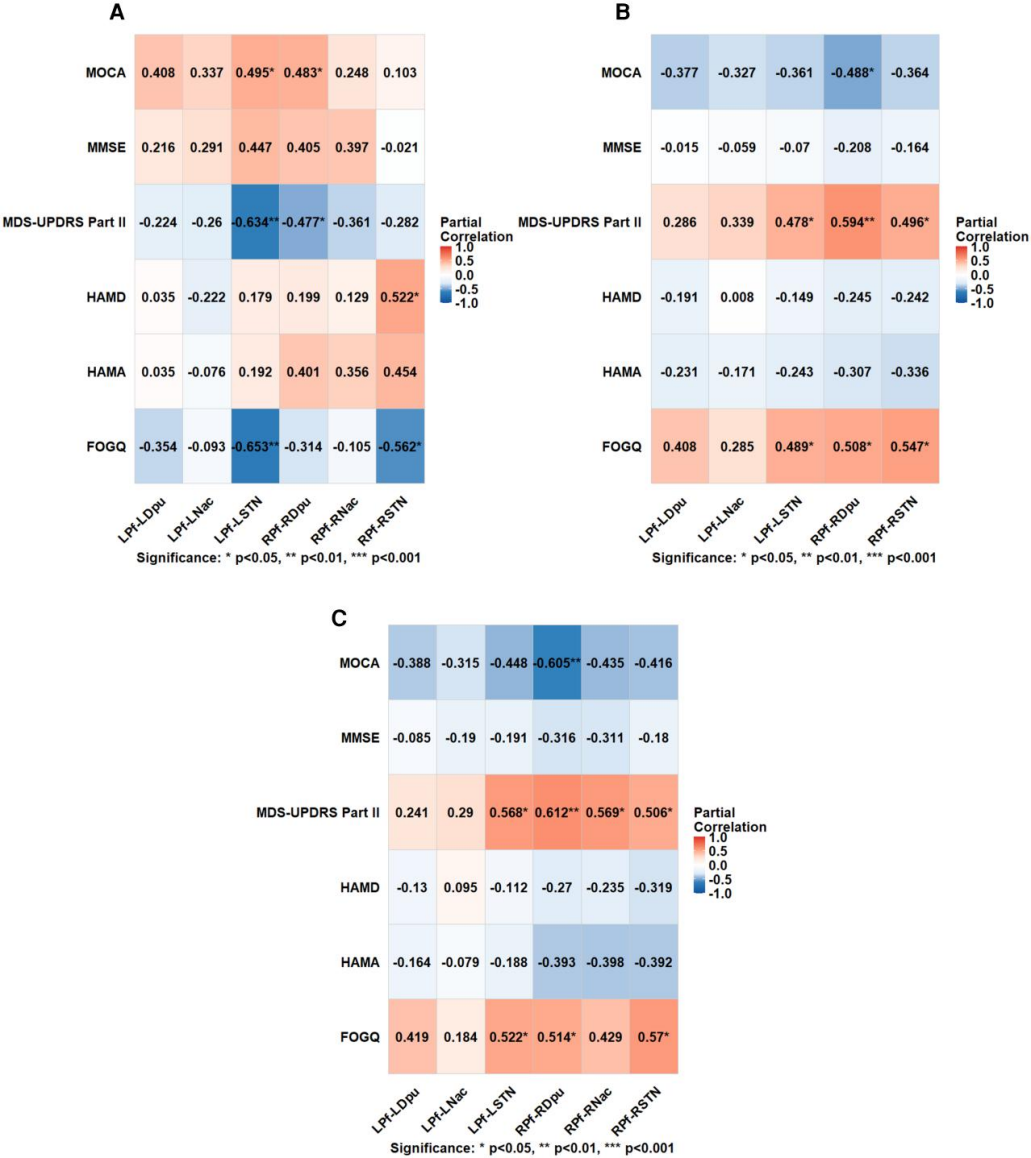
**Partial correlations between imaging metrics and clinical measures in the PIGD-FOG subgroup (*n* = 21).** Partial correlations between clinical measures and FA values in Pf projections in the PIGD-FOG subgroup (**A**), MD values in Pf projections in the PIGD-FOG subgroup (**B**) and RD values in Pf projections in the PIGD-FOG subgroup (**C**). LPf, left parafascicular nucleus; LDpu, left dorsal putamen; LNac, left nucleus accumbens; LSTN, left subthalamic nucleus; RPf, right parafascicular nucleus; RDpu, right dorsal putamen; RNac, right nucleus accumbens; RSTN, right subthalamic nucleus; L, left; R, right; MDS-UPDRS Part II, Movement Disorder Society Unified Parkinson’s Disease Rating Scale, Part II; MOCA, Montreal Cognitive Assessment; MMSE, Mini-Mental State Examination; HAMD, Hamilton Depression Scale; HAMA, Hamilton Anxiety Scale; FOGQ, Freezing of Gait Questionnaire. Significance: *P < 0.05, **P < 0.01; ***P < 0.001.

In the PIGD-FOG subgroup, FOGQ scores were significantly negatively correlated with FA values in the bilateral Pf-STN pathways (left, *r* = −0.653, *P* = 0.003; right, *r* = −0.562, *P* = 0.015). FA values in the left Pf-STN pathway and right Pf-Dpu pathway were positively correlated with MOCA scores (*r* = 0.495, *P* = 0.037; *r* = 0.483, *P* = 0.042, respectively) and negatively correlated with MDS-UPDRS Part II scores (*r* = −0.634, *P* = 0.005; *r* = −0.477, *P* = 0.046, respectively). In addition, FA value in the right Pf-STN pathway was positively correlated with HAMD scores (*r* = 0.522, *P* = 0.026). Regarding MD metrics, FOGQ scores were positively correlated with MD values in the bilateral Pf-STN pathways (left, *r* = 0.489, *P* = 0.040; right, *r* = 0.547, *P* = 0.019) and right Pf-Dpu pathway (*r* = 0.508, *P* = 0.032). MD value in the right Pf-Dpu pathway was negatively correlated with MOCA scores (*r* = −0.488, *P* = 0.040). Moreover, MDS-UPDRS Part II scores were positively correlated with MD values in the bilateral Pf-STN pathways (left, *r* = 0.478, *P* = 0.045; right, *r* = 0.496, *P* = 0.036) and right Pf-Dpu pathway (*r* = 0.594, *P* = 0.009). Similarly, regarding RD metrics, FOGQ scores were positively correlated with RD values in the bilateral Pf-STN pathways (left, *r* = 0.522, *P* = 0.017; right, *r* = 0.570, *P* = 0.014) and right Pf-Dpu pathway (*r* = 0.514, *P* = 0.029). RD value in the right Pf-Dpu pathway was negatively correlated with MOCA scores (*r* = −0.605, *P* = 0.008). Furthermore, MDS-UPDRS Part II scores were positively correlated with RD values in the bilateral Pf-STN pathways (left, *r* = 0.568, *P* = 0.014; right, *r* = 0.506, *P* = 0.032), right Pf-Dpu pathway (*r* = 0.612, *P* = 0.007) and right Pf-Nac pathway (*r* = 0.569, *P* = 0.014). The details are presented in Fig. 6. No other diffusion metrics showed significant correlations with clinical measures in this subgroup. These results suggested that the microstructural alterations of these pathways were associated with FOG severity, cognitive impairment and greater impairment in motor-related activities of daily living in the PIGD-FOG subgroup. Additionally, partial correlations were performed to explore the relationships between the PDTD subtype and clinical characteristics, with detailed results provided in Supplementary Fig. 2.

## Discussion

Our study is, to our knowledge, the first demonstration on the microstructural alterations of Pf projections across Parkinson’s disease motor subtypes, with a specific focus on PIGD patients with FOG, by using both DTI and NODDI. The major findings of this study were (i) PIGD subtype exhibited microstructural degeneration of Pf pathways demonstrating reduced FA and NDI values, along with increased AD, MD and RD values compared with HC, whereas no significant differences were observed between the PDTD and HC groups or between the PDTD and PIGD groups. (ii) Within the PIGD subtype, patients with FOG exhibited more severe degeneration of Pf pathways than those without FOG, and these alterations were significantly associated with FOG severity, cognitive impairment and greater impairment in motor-related activities of daily living. (iii) Microstructural metrics in Pf pathways exhibited remarkable discriminative performance in distinguishing PIGD-FOG from PIGD-nFOG. (iv) Microstructural impairments of Pf pathways in the PIGD subtype were associated with more severe gait dysfunction, more advanced disease stage and greater impairment in motor-related activities of daily living. Collectively, these findings indicate that microstructural alterations of Pf pathways are preferentially associated with the PIGD subtype and are more pronounced in patients with FOG, underscoring a strong association between Pf-related circuits and FOG in PIGD.

### Microstructural impairments of Pf pathways in the PIGD subtype

In the present study, we demonstrated microstructural degeneration of Pf pathways in the PIGD subtype. Degeneration of the Pf-STN pathway may be associated with α-synuclein deposition. In Parkinson’s disease, the spread of α-synuclein through neural circuits and synapses led to neuronal loss and disruption of connecting WM pathways.^41^ Early studies have reported significant neuron loss in the centre-median/parafascicular (CM/Pf) complex, with increased α-synuclein deposition associated with greater atrophy in this area.^42,43^ Mechanistically, α-synuclein changed the levels of proteins involved in axonal transport, leading to abnormal accumulation of proteins in the cell body and subsequent axonal transport deficits,^44^ which may ultimately result in microstructural abnormalities in the Pf-STN pathway. In addition, damage to this pathway may also be associated with a reduction in parvalbumin-positive (PV^+^) neurons. Previous studies have shown that the Pf region contains many PV^+^ neurons, which are significantly depleted in Parkinson’s disease.^43^ Notably, a substantial proportion of Pf neurons projected to PV ^+^ STN neurons within the STN,^8^ suggesting that the Pf-STN pathway exhibited significant neuron loss. Consistent with these pathological observations, our study revealed reduced FA and NDI values, along with increased AD, MD and RD values in the Pf-STN pathways of PIGD patients compared with HC, suggesting axonal loss and/or microstructural disorganization within these fibre bundles.^14,45-47^ Moreover, inhibition of these PV ^+^ STN neurons impaired motor learning abilities.^8^ Animal studies demonstrated that Pf-defined STN efferents terminated in various BG output nuclei, including the SNr, the globus pallidus internus (GPi) and other critical brainstem regions involved in motor control, particularly gait regulation.^48^ The BG output nuclei, especially the SNr, projected to the mesencephalic locomotor region (MLR), which played a key role in regulating subconscious aspects of muscle tone, postural balance and gait control.^49^ Disruption of this pathway may lead to desynchronization of STN activity and impair both the indirect and hyperdirect BG pathways, resulting in worsened motor disruption, especially gait disturbances such as FOG. Consistent with this view, in the PIGD subtype, we found that microstructural degeneration of bilateral Pf-STN pathways was associated with more severe gait dysfunction and more advanced disease stage. Moreover, PIGD-FOG patients exhibited more severe degeneration of bilateral Pf-STN pathways compared with those without FOG. Together, these findings underscore a critical role of the Pf-STN pathway in locomotor control and gait regulation. Notably, we observed positive correlations between NDI values in the left Pf-Dpu pathway and left Pf-STN pathway and disease duration in PIGD patients. This finding may reflect a compensatory mechanism, especially given that the overall NDI values in PIGD patients remained lower than those observed in HC. In line with this interpretation, a previous study has suggested that increased NDI values may be associated with iron deposition and/or reactive astrogliosis in the affected regions.^50^ In addition, long-term overdose of levodopa treatment might also contribute to elevated NDI values in Parkinson’s disease patients with gait disorders.^51,52^

We also observed reduced FA and NDI values, along with increased AD and MD values in the Pf-Dpu pathways of PIGD patients, indicating pronounced microstructural degeneration. Correlation analyses further demonstrated that greater microstructural damage was associated with more severe gait dysfunction and impairment in motor-related activities of daily living, as reflected by higher gait and MDS-UPDRS Part II scores. Consistent with these findings, PIGD-FOG patients exhibited significantly greater microstructural degeneration within the Pf-Dpu pathways compared with PIGD-nFOG patients. However, Chen *et al*.^9^ reported a positive association between effective connectivity (EC) from the Pf to the Dpu and motor severity as measured by MDS-UPDRS Part III scores. In contrast, in our study, the microstructural integrity of Pf pathways showed no significant association with MDS-UPDRS Part III scores. Similarly, Lenfeldt *et al*.^12^ also reported no significant associations between diffusion metrics in the thalamus and BG and MDS-UPDRS Part III, which they attributed to medication effects. Even after including LEDD as an additional covariate, no significant association with MDS-UPDRS Part III was observed in our analyses (Supplementary Table 6). These findings suggest that Pf-related microstructural alterations may be more closely associated with axial and gait-related symptoms than with overall motor severity. In our study, the microstructural integrity of Pf pathways showed a negative correlation with gait disturbance. Interestingly, as previously reported, Chen *et al*.^9^ observed an opposite direction of association. This discrepancy may reflect stage-dependent compensatory mechanisms that attempt to counteract progressive disease-related changes. The Pf was considered capable of exerting a facilitatory influence on dopaminergic (DA) transmission in the caudate putamen (CP). Both dopamine D1 and D2 receptor-expressing neurons in the CP received inputs from the Pf.^8^ Moreover, unilateral electrical stimulation of the Pf in halothane-anaesthetized rats resulted in bilateral elevation of DA utilization in the CP.^53^ In Chen *et al*.,^9^ patients were in the early stages of the disease (H&Y < 2.5), during which Pf-Dpu connectivity may be upregulated to enhance DA transmission as a compensatory response to emerging dopaminergic dysfunction. In contrast, patients in our study were in the middle stage of the disease (H&Y > 2.5), characterized by more severe clinical symptoms and greater microstructural damage. At this stage, compensatory mechanisms may become insufficient to counteract the damage.

Interestingly, microstructural alterations of Pf pathways were significantly associated with MDS-UPDRS Part II scores in the PIGD subtype. This relationship may reflect the impact of degeneration in Pf pathways on axial motor symptoms, including gait impairments and FOG, which significantly affect activities of daily living. A systematic review and meta-analysis demonstrated a moderate association between gait disturbances and activity limitations in patients with idiopathic Parkinson’s disease,^54^ and multiple studies showed that the presence of FOG was associated with poorer quality of life.^55,56^ Moreover, the relatively high prevalence of FOG in our cohort may have further strengthened this association, as FOG is known to significantly impair functional mobility and activities of daily living. Consistent with these observations, we also found significant associations between microstructural metrics in Pf pathways and MDS-UPDRS Part II scores in the PIGD-FOG subgroup. This further suggests that the association between microstructural degeneration of Pf pathways and MDS-UPDRS Part II scores may be related to the impact of gait impairments and FOG on activities of daily living. Given the limited sample size in the current study, future research with larger cohorts is needed to determine whether the association between Pf-related microstructural alterations and impairments in daily living is mediated by gait dysfunction and FOG.

In the Pf-Nac pathway, we observed that the AD value in the left Pf-Nac tract was significantly higher in PIGD compared to HC. This alteration may be associated with the influence of DA stimulation on the mesolimbic system. Both D1 and D2 neurons in Nac received Pf inputs, similar to D1/D2 neurons in CP.^8^ The presence of a strong DA influence on the mesolimbic pathway, which includes both the hippocampus and the Nac, has been established in both physiological and pathological settings.^57^ Previous studies have linked the Pf-Nac pathway to affective processing, reporting its association with the severity of depression.^9,58^ However, in our study, no significant association was observed between the integrity of this tract and depression severity. This discrepancy may reflect the fact that depression in Parkinson’s disease is not solely related to dopaminergic dysfunction but also involves dysregulation of broader monoaminergic systems. Supporting this, Xu *et al*.^59^ reported that alterations in monoaminergic neurotransmitter expression were associated with abnormal functional connectivity in the striatum-mesolimbic circuit. Moreover, they observed a significant reduction in vesicular monoamine transporter 2 (VMAT2) binding in the Nac of depressed Parkinson’s disease patients, which was significantly correlated with depression severity. Together, these findings indicate that microstructural alterations of the Pf-Nac pathway may be linked to mesolimbic dopaminergic modulation, but they are insufficient to fully explain depression in Parkinson’s disease.

Group comparisons revealed significant differences between the PIGD and HC groups, whereas no significant differences were observed between the PDTD and HC groups or between the PDTD and PIGD groups. This result is partially supported by previous studies, which reported no differences in FA or MD at the whole-brain level using Tract-Based Spatial Statistics (TBSS) between PDTD and PIGD or between PIGD and HC.^13,60^ Relative to HC, the PDTD subtype exhibited a trend of decreased FA and NDI, alongside increased AD, MD and RD values in Pf projections, although these differences did not reach statistical significance. This pattern may reflect the relatively benign nature of the PDTD subtype, as supported by lower H&Y stage, MDS-UPDRS Part II and III scores compared with the PIGD subtype in our study. Consistent with this interpretation, prior post-mortem studies have reported less neuronal cell loss in both dopaminergic and non-dopaminergic neural circuits in PDTD compared with non-tremor Parkinson’s disease patients.^61,62^ Furthermore, this may reflect differences in motor circuits between subtypes, as AD values in the bilateral Pf-STN pathways and the left Pf-Nac pathway were negatively correlated with tremor scores (Supplementary Fig. 2). In addition, heterogeneity within the PIGD subgroup may have contributed to the absence of significant differences between PDTD and the overall PIGD group. When comparing the PDTD and PIGD-nFOG groups, FA values in Pf pathways were significantly higher in PIGD-nFOG patients than in PDTD patients. In contrast, at the overall PIGD level, FA values tended to be lower than those in the PDTD group, although this difference did not reach statistical significance. Together, these findings suggest that microstructural alterations of Pf-related pathways are more pronounced in PIGD patients with prominent axial and gait dysfunction, particularly in those with FOG. These results also highlight the importance of considering symptom-specific clinical measures when investigating the neural correlates of Parkinson’s disease motor subtypes.

### Microstructural alterations of Pf pathways associated with FOG

After controlling for MDS-UPDRS Part III scores, microstructural alterations of Pf pathways remained significantly different between PIGD subgroups, indicating that these differences were not solely driven by differences in overall disease severity but also by the presence of FOG. Within the PIGD-FOG subgroup, microstructural metrics in Pf pathways were associated with FOG severity and effectively differentiated patients with and without FOG, highlighting the close link between microstructural integrity of Pf pathways and FOG. Microstructural metrics in Pf pathways showed strong discriminative performance in differentiating PIGD-FOG from PIGD-nFOG (all AUCs > 0.8). Among these models, the Pf-STN-based model showed the highest AUC numerically (AUC = 0.927), with a sensitivity of 81.0% and specificity of 92.3%, although this difference did not reach statistical significance compared with the other Pf pathway-based models. Similarly, the Pf-STN-based model also demonstrated strong discriminative performance in differentiating PIGD-FOG patients from HC, with an AUC of 0.959, 95.2% sensitivity and 84.2% specificity (Supplementary Fig. 3 and Supplementary Table 7). Together, these findings suggest that microstructural metrics in Pf pathways may serve as reliable markers for identifying FOG-related phenotypes, while maintaining a low false-positive rate. However, further studies are needed to validate these findings in larger cohorts.

As previously discussed, FA values in the Pf pathways were significantly higher in PIGD-nFOG patients than in PDTD patients. However, when considering the PIGD group as a whole, FA values tended to be lower than those in the PDTD group, although this difference did not reach statistical significance. This relative increase in FA observed in PIGD-nFOG patients may reflect compensatory or adaptive microstructural reorganization associated with preserved gait function, rather than indicating reduced disease burden. According to the threshold model hypothesis proposed by Plotnik *et al*.,^63^ FOG occurs when gait impairments accumulate beyond a critical threshold, leading to motor breakdown. PIGD-nFOG patients may represent an intermediate stage in which compensatory mechanisms are still partially effective, whereas PIGD-FOG patients have surpassed this threshold. Consistent with this hypothesis, PIGD-FOG patients in our cohort exhibited more severe clinical impairments, as reflected by higher gait performance scores, FOGQ scores, MDS-UPDRS Parts II and III scores and more advanced H&Y stage. Furthermore, microstructural degeneration of Pf pathways was associated with more severe gait dysfunction and more advanced disease stage in the overall PIGD group, as well as with greater FOG severity within the PIGD-FOG subgroup. Overall, our findings demonstrate that microstructural alterations of Pf pathways are more closely linked to FOG, providing further support for the threshold model hypothesis, in which gait impairments beyond a critical threshold trigger freezing episodes.

Given that motor symptom laterality is a hallmark of Parkinson’s disease and has been associated with disease progression, we examined whether laterality influenced the observed findings. Chen *et al*.^64^ reported that patients with the left akinetic/rigid dominant (LAR) subtype exhibited more severe motor manifestations and greater grey and WM damage. Consistent with this observation, 17 of 21 PIGD-FOG patients in our cohort were classified as left-dominant Parkinson’s disease. In addition, Fling *et al*.^65^ reported laterality effects in FOG, with structural differences observed solely in the right hemisphere of patients with FOG. In our study, after controlling for motor symptom laterality, significant microstructural alterations in the Pf-Dpu and Pf-STN pathways were observed between PIGD-FOG and PIGD-nFOG groups. This suggests that FOG-related microstructural changes may be partially masked by motor asymmetry if laterality is not accounted for. Therefore, adjusting for laterality may help to reveal microstructural alterations specifically associated with FOG, rather than reflecting asymmetric motor deficits. Importantly, the overall pattern of results remained unchanged, supporting the robustness of the main findings.

Regarding non-dopaminergic contributions to gait dysfunction, the cholinergic system has been shown to play a critical role in the regulation of balance and gait in Parkinson’s disease.^66,67^ Emerging evidence further suggests that cholinergic dysfunction contributes to impaired gait control and postural instability and plays a key role in FOG.^68,69^ Two major sources of cholinergic projections in the brain are the nucleus basalis of Meynert (NBM) and the pedunculopontine nucleus (PPN). Gan *et al*.^70^ found that FOG was linked to atrophy in the NBM, the severity of which correlated with progressive deterioration of FOG. Beyond the NBM, the PPN, the major component of the MLR, is another key cholinergic structure involved in Parkinson’s disease. The PPN contributes to both motor and non-motor functions, including arousal, attention and learning.^71^ Functionally, it acts as a transit station in locomotor control, receiving motor commands from upstream motor regions and sending both ascending and descending projections to motor areas.^72^ Degeneration of the PPN has been implicated in axial motor symptoms of Parkinson’s disease, including postural and gait impairments, as well as FOG.^73-75^ DBS targeting the PPN has emerged as a treatment for FOG relief for some patients.^76^ Anatomical and physiological studies have shown that the Pf receives strong cholinergic projections from the PPN.^77-79^ Consistent with this, an electrophysiological study demonstrated decreased activity of Pf neurons after PPN lesions, highlighting the significant influence of the PPN on Pf neuronal activity.^80^ The relationship between Pf pathways and gait function may be related to the role of the PPN-Pf circuit in gait regulation. Microstructural damage to the Pf pathways could disrupt this circuit, resulting in severe gait impairments and FOG. In addition, acetylcholine receptors (AChRs) are highly expressed in neurons within Pf-related pathways,^8,81^ further highlighting the involvement of the cholinergic system in these Pf pathways. Beyond gait-related functions, the cholinergic system, particularly the NBM, also plays a critical role in cognitive function in Parkinson’s disease. Previous studies have demonstrated that microstructural degeneration of NBM tracts is associated with cognitive impairments in Parkinson’s disease.^82^ Consistent with these findings, we observed significant correlations between microstructural degeneration of Pf pathways and MOCA scores in the PIGD-FOG subgroup. However, in the overall PIGD group, no significant associations were observed between microstructural measures and cognitive performance. This pattern further emphasizes the association between the cholinergic system and FOG, suggesting that cholinergic-related microstructural damage may be more pronounced in patients with severe gait impairments, such as FOG. In summary, these results further emphasize the involvement of the cholinergic system in the Pf pathways and its association with FOG.

Several limitations of this study should be considered. First, the relatively small sample size within the PIGD subgroups, particularly given the high AUC values observed in the ROC models, may limit the generalizability of our findings. Future studies should implement cross-validation and external validation using independent cohorts to assess model robustness and reproducibility. Second, the cross-sectional design limits the ability to assess longitudinal changes in the microstructural integrity of Pf pathways, highlighting the need for longitudinal studies to track disease progression. Third, the extended DWI scan duration limits the sample size, and larger cohorts will be essential for further investigating the discriminative and mediating roles of Pf-related alterations using logistic regression and mediation models. Finally, our study focused on the PIGD-FOG subgroup without differentiating OFF and ON medication states. Thus, it remains unclear whether the observed microstructural alterations of Pf pathways are specifically associated with FOG during the OFF or ON medication state. Future studies incorporating state-dependent FOG assessments are needed to clarify this relationship.

## Conclusion

In conclusion, this study indicates that microstructural alterations of Pf pathways are preferentially associated with the PIGD subtype and are more pronounced in patients with FOG, underscoring a strong association between Pf-related circuits and FOG in PIGD. These findings highlight the importance of symptom-driven heterogeneity within motor subtypes and provide a refined perspective for Parkinson’s disease classification, as well as for future clinical stratification aimed at improving behavioural outcomes.

## Supplementary Material

fcag154_Supplementary_Data

## Data Availability

The data that support the findings of this study are available from the corresponding author upon reasonable request.
