# Supplementary material for: Microstructural alterations of parafascicular nucleus pathways associated with freezing of gait in Parkinson’s disease
Source: Brain Commun. 2026 May 20;8(3):fcag154. doi: 10.1093/braincomms/fcag154 (PMC13190275; doi:10.1093/braincomms/fcag154)
Supplement: fcag154_Supplementary_Data [file fcag154_supplementary_data.docx]

**Supplementary material**

**Supplementary material 1. Criteria for classifying motor symptom laterality**

The MDS-UPDRS Part III scores for items 3.3-3.8 were summed separately for the right and left limbs to calculate right-side and left-side scores. Based on these scores, PIGD-FOG and PIGD-nFOG patients were categorized into left-dominant PD (LPD) or right-dominant PD (RPD). The laterality index (LI) was defined by subtracting the left-side scores from the right-side scores. A positive LI indicated RPD patients, and a negative one represented LPD patients.^[1]^ Motor symptom laterality was subsequently modeled as a categorical covariate (RPD vs. LPD), based on the sign of the LI, and included in the statistical model to assess its potential influence on the results.

**Supplementary Table 1A. DTI changes among HC and PD subtypes**

| DTI metrics | pathways | HCs(n = 38) | PD(n = 59) | | P-value | P-value(post hoc test) | effect size(partial η^2^) |
| --- | --- | --- | --- | --- | --- | --- | --- |
|  |  |  | PDTD(n = 25) | PIGD(n = 34) |  |  |  |
| FA×10^-1^ | LPf-LDpu | 4.770±0.191 | 4.681±0.153 | 4.635±0.227 | 0.071 | P_1_=0.368 | 0.056 |
|  |  |  |  |  |  | P_2_=0.073 |  |
|  |  |  |  |  |  | P_3_=1.000 |  |
|  | LPf-LNac | 4.417±0.239 | 4.303±0.169 | 4.254±0.251 | 0.224 | P_1_=0.655 | 0.032 |
|  |  |  |  |  |  | P_2_=0.284 |  |
|  |  |  |  |  |  | P_3_=1.000 |  |
|  | LPf-LSTN | 4.976(0.328) | 4.905(0.198) | 4.744(0.358) | **0.005** | P_1_=0.353 | 0.111 |
|  |  |  |  |  |  | P**_2_=0.003** |  |
|  |  |  |  |  |  | P_3_=0.241 |  |
|  | RPf-RDpu | 4.734±0.196 | 4.649±0.168 | 4.614±0.186 | **0.032** | P_1_=0.195 | 0.073 |
|  |  |  |  |  |  | P**_2_=0.032** |  |
|  |  |  |  |  |  | P_3_=1.000 |  |
|  | RPf-RNac | 4.357(0.293) | 4.285(0.271) | 4.157(0.220) | 0.120 | P_1_=0.617 | 0.045 |
|  |  |  |  |  |  | P_2_=0.125 |  |
|  |  |  |  |  |  | P_3_=1.000 |  |
|  | RPf-RSTN | 4.979±0.191 | 4.906±0.180 | 4.796±0.201 | **0.005** | P_1_=0.588 | 0.108 |
|  |  |  |  |  |  | P**_2_=0.004** |  |
|  |  |  |  |  |  | P_3_=0.152 |  |
| AD×10^-4^ | LPf-LDpu | 8.337(0.281) | 8.473(0.317) | 8.635(0.345) | **0.016** | P1=1.000 | 0.086 |
|  |  |  |  |  |  | P**_2_=0.015** |  |
|  |  |  |  |  |  | P_3_=0.170 |  |
|  | LPf-LNac | 8.536±0.176 | 8.619±0.195 | 8.716±0.196 | **0.026** | P_1_=0.885 | 0.077 |
|  |  |  |  |  |  | P**_2_=0.023** |  |
|  |  |  |  |  |  | P_3_=0.296 |  |
|  | LPf-LSTN | 8.365±0.203 | 8.531±0.248 | 8.635±0.220 | **0.009** | P_1_=0.194 | 0.099 |
|  |  |  |  |  |  | P**_2_=0.007** |  |
|  |  |  |  |  |  | P_3_=0.634 |  |
|  | RPf-RDpu | 8.311±0.226 | 8.424±0.235 | 8.554±0.290 | 0.059 | P_1_=1.000 | 0.060 |
|  |  |  |  |  |  | P_2_=0.058 |  |
|  |  |  |  |  |  | P_3_=0.420 |  |
|  | RPf-RNac | 8.546±0.179 | 8.599±0.232 | 8.689±0.238 | 0.157 | P_1_=1.000 | 0.040 |
|  |  |  |  |  |  | P_2_=0.189 |  |
|  |  |  |  |  |  | P_3_=0.555 |  |
|  | RPf-RSTN | 8.368±0.207 | 8.508±0.226 | 8.588±0.238 | **0.043** | P_1_=0.351 | 0.067 |
|  |  |  |  |  |  | P**_2_=0.039** |  |
|  |  |  |  |  |  | P_3_=1.000 |  |
| MD×10^-4^ | LPf-LDpu | 5.443(0.237) | 5.558(0.235) | 5.667(0.291) | **0.048** | P_1_=0.729 | 0.065 |
|  |  |  |  |  |  | P**_2_=0.042** |  |
|  |  |  |  |  |  | P_3_=0.576 |  |
|  | LPf-LNac | 5.681(0.278) | 5.805(0.261) | 5.889(0.261) | 0.099 | P_1_=0.573 | 0.050 |
|  |  |  |  |  |  | P_2_=0.099 |  |
|  |  |  |  |  |  | P_3_=1.000 |  |
|  | LPf-LSTN | 5.308±0.209 | 5.463±0.201 | 5.552±0.195 | **0.006** | P_1_=0.129 | 0.106 |
|  |  |  |  |  |  | P**_2_=0.005** |  |
|  |  |  |  |  |  | P_3_=0.729 |  |
|  | RPf-RDpu | 5.371(0.239) | 5.462(0.192) | 5.625(0.271) | 0.054 | P_1_=0.814 | 0.062 |
|  |  |  |  |  |  | P_2_=0.049 |  |
|  |  |  |  |  |  | P_3_=0.563 |  |
|  | RPf-RNac | 5.685(0.223) | 5.810(0.228) | 5.942(0.217) | 0.175 | P_1_=0.772 | 0.038 |
|  |  |  |  |  |  | P_2_=0.193 |  |
|  |  |  |  |  |  | P_3_=1.000 |  |
|  | RPf-RSTN | 5.293(0.259) | 5.395(0.206) | 5.495(0.292) | **0.045** | P_1_=0.629 | 0.066 |
|  |  |  |  |  |  | P**_2_=0.040** |  |
|  |  |  |  |  |  | P_3_=0.642 |  |
| RD×10^-4^ | LPf-LDpu | 3.973(0.254) | 4.070(0.275) | 4.181(0.318) | 0.146 | P_1_=0.781 | 0.041 |
|  |  |  |  |  |  | P_2_=0.153 |  |
|  |  |  |  |  |  | P_3_=1.000 |  |
|  | LPf-LNac | 4.254(0.332) | 4.370(0.298) | 4.473(0.302) | 0.226 | P_1_=0.655 | 0.032 |
|  |  |  |  |  |  | P_2_=0.287 |  |
|  |  |  |  |  |  | P_3_=1.000 |  |
|  | LPf-LSTN | 3.772(0.347) | 3.891(0.246) | 4.054(0.268) | **0.022** | P_1_=0.216 | 0.081 |
|  |  |  |  |  |  | P**_2_=0.019** |  |
|  |  |  |  |  |  | P_3_=1.000 |  |
|  | RPf-RDpu | 3.946(0.241) | 4.022(0.174) | 4.173(0.247) | 0.109 | P_1_=0.743 | 0.048 |
|  |  |  |  |  |  | P_2_=0.108 |  |
|  |  |  |  |  |  | P_3_=1.000 |  |
|  | RPf-RNac | 4.280(0.306) | 4.388(0.238) | 4.522(0.222) | 0.243 | P_1_=0.643 | 0.031 |
|  |  |  |  |  |  | P_2_=0.324 |  |
|  |  |  |  |  |  | P_3_=1.000 |  |
|  | RPf-RSTN | 3.754(0.326) | 3.846(0.223) | 3.941(0.299) | 0.106 | P_1_=1.000 | 0.048 |
|  |  |  |  |  |  | P_2_=0.105 |  |
|  |  |  |  |  |  | P_3_=0.740 |  |

Continuous variables are expressed as median (interquartile range-IQR) for non-normally distributed data and as mean ± standard deviation for normally distributed data.

DTI metrics in the Pf pathways are compared among PD motor subtypes and HC using general linear models, controlling for sex, age, and years of education. Bold values indicate statistically significant differences between groups (P < 0.05).

P_1_, Significant differences between HC and PDTD, Bonferroni corrected;

P_2_, Significant differences between HC and PIGD, Bonferroni corrected;

P_3_, Significant differences between PDTD and PIGD, Bonferroni corrected.

**Supplementary Table 1B. NODDI changes among HC and PD subtypes**

| NODDI metrics | pathways | HC(n = 38) | PD(n = 59) | | P-value | P-value(post hoc test) | effect size(partial η^2^) |
| --- | --- | --- | --- | --- | --- | --- | --- |
|  |  |  | PDTD(n = 25) | PIGD(n = 34) |  |  |  |
| NDI×10^-1^ | LPf-LDpu | 6.363(0.501) | 6.161(0.443) | 6.050(0.608) | **0.034** | P_1_=0.422 | 0.071 |
|  |  |  |  |  |  | **P_2_=0.029** |  |
|  |  |  |  |  |  | P_3_=0.777 |  |
|  | LPf-LNac | 6.099(0.512) | 5.859(0.472) | 5.824(0.535) | 0.108 | P_1_=0.596 | 0.048 |
|  |  |  |  |  |  | P_2_=0.109 |  |
|  |  |  |  |  |  | P_3_=1.000 |  |
|  | LPf-LSTN | 6.604±0.403 | 6.346±0.348 | 6.194±0.352 | **0.019** | P_1_=0.242 | 0.084 |
|  |  |  |  |  |  | P**_2_=0.016** |  |
|  |  |  |  |  |  | P_3_=0.861 |  |
|  | RPf-RDpu | 6.497(0.487) | 6.301(0.472) | 6.037(0.575) | **0.036** | P_1_=0.533 | 0.071 |
|  |  |  |  |  |  | P**_2_=0.030** |  |
|  |  |  |  |  |  | P_3_=0.639 |  |
|  | RPf-RNac | 6.073(0.434) | 5.901(0.373) | 5.752(0.416) | 0.088 | P_1_=0.378 | 0.052 |
|  |  |  |  |  |  | P_2_=0.097 |  |
|  |  |  |  |  |  | P_3_=1.000 |  |
|  | RPf-RSTN | 6.708(0.478) | 6.484(0.346) | 6.273(0.439) | **0.032** | P_1_=0.486 | 0.073 |
|  |  |  |  |  |  | P**_2_=0.027** |  |
|  |  |  |  |  |  | P_3_=0.647 |  |
| ODI×10^-1^ | LPf-LDpu | 2.690(0.168) | 2.700(0.124) | 2.656(0.211) | 0.646 | P_1_=1.000 | 0.010 |
|  |  |  |  |  |  | P_2_=1.000 |  |
|  |  |  |  |  |  | P_3_=1.000 |  |
|  | LPf-LNac | 2.801(0.209) | 2.824(0.206) | 2.838(0.258) | 0.752 | P_1_=1.000 | 0.006 |
|  |  |  |  |  |  | P_2_=1.000 |  |
|  |  |  |  |  |  | P_3_=1.000 |  |
|  | LPf-LSTN | 2.581(0.156) | 2.583(0.154) | 2.634(0.215) | 0.877 | P_1_=1.000 | 0.003 |
|  |  |  |  |  |  | P_2_=1.000 |  |
|  |  |  |  |  |  | P_3_=1.000 |  |
|  | RPf-RDpu | 2.715(0.162) | 2.725(0.214) | 2.745(0.269) | 0.800 | P_1_=1.000 | 0.005 |
|  |  |  |  |  |  | P_2_=1.000 |  |
|  |  |  |  |  |  | P_3_=1.000 |  |
|  | RPf-RNac | 2.867(0.171) | 2.895(0.211) | 2.982(0.270) | 0.929 | P_1_=1.000 | 0.002 |
|  |  |  |  |  |  | P_2_=1.000 |  |
|  |  |  |  |  |  | P_3_=1.000 |  |
|  | RPf-RSTN | 2.599(0.148) | 2.591(0.195) | 2.604(0.194) | 0.974 | P_1_=1.000 | <0.001 |
|  |  |  |  |  |  | P_2_=1.000 |  |
|  |  |  |  |  |  | P_3_=1.000 |  |
| FWF×10^-1^ | LPf-LDpu | 3.451(0.255) | 3.323(0.324) | 3.314(0.287) | 0.626 | P_1_=1.000 | 0.010 |
|  |  |  |  |  |  | P_2_=1.000 |  |
|  |  |  |  |  |  | P_3_=1.000 |  |
|  | LPf-LNac | 3.621(0.283) | 3.575(0.343) | 3.621(0.329) | 0.987 | P_1_=1.000 | <0.001 |
|  |  |  |  |  |  | P_2_=1.000 |  |
|  |  |  |  |  |  | P_3_=1.000 |  |
|  | LPf-LSTN | 3.369(0.188) | 3.387(0.243) | 3.387(0.288) | 0.940 | P_1_=1.000 | 0.001 |
|  |  |  |  |  |  | P_2_=1.000 |  |
|  |  |  |  |  |  | P_3_=1.000 |  |
|  | RPf-RDpu | 3.505(0.237) | 3.424(0.280) | 3.504(0.255) | 0.888 | P_1_=1.000 | 0.003 |
|  |  |  |  |  |  | P_2_=1.000 |  |
|  |  |  |  |  |  | P_3_=1.000 |  |
|  | RPf-RNac | 3.684(0.223) | 3.655(0.401) | 3.846(0.348) | 0.525 | P_1_=1.000 | 0.014 |
|  |  |  |  |  |  | P_2_=1.000 |  |
|  |  |  |  |  |  | P_3_=0.826 |  |
|  | RPf-RSTN | 3.453(0.235) | 3.375(0.240) | 3.499(0.287) | 0.736 | P_1_=1.000 | 0.007 |
|  |  |  |  |  |  | P_2_=1.000 |  |
|  |  |  |  |  |  | P_3_=1.000 |  |

Continuous variables are expressed as median (interquartile range-IQR) for non-normally distributed data and as mean ± standard deviation for normally distributed data.

NODDI metrics in the Pf pathways are compared among PD motor subtypes and HC using general linear models, controlling for sex, age, and years of education. Bold values indicate statistically significant differences between groups (P < 0.05).

P_1_, Significant differences between HC and PDTD, Bonferroni corrected;

P_2_, Significant differences between HC and PIGD, Bonferroni corrected;

P_3_, Significant differences between PDTD and PIGD, Bonferroni corrected.

**Supplementary Table 2A.** **DTI changes in PIGD patients with and without FOG**

| DTI metrics | pathways | PIGD-nFOG(n = 13) | PIGD-FOG(n = 21) | P-value | effect size(partial η^2^) |
| --- | --- | --- | --- | --- | --- |
| FA×10^-1^ | LPf-LDpu | 4.777±0.210 | 4.548±0.193 | **0.022** | 0.168 |
|  |  |  |  |  |  |
|  | LPf-LNac | 4.404±0.223 | 4.161±0.225 | **0.012** | 0.197 |
|  |  |  |  |  |  |
|  | LPf-LSTN | 4.932±0.211 | 4.633±0.181 | **0.002** | 0.285 |
|  |  |  |  |  |  |
|  | RPf-RDpu | 4.730±0.170 | 4.542±0.160 | **0.006** | 0.236 |
|  |  |  |  |  |  |
|  | RPf-RNac | 4.237(0.230) | 4.106(0.253) | **0.004** | 0.255 |
|  |  |  |  |  |  |
|  | RPf-RSTN | 4.960±0.156 | 4.694±0.154 | **<0.001** | 0.374 |
|  |  |  |  |  |  |
| AD×10^-4^ | LPf-LDpu | 8.556±0.194 | 8.709±0.247 | 0.065 | 0.112 |
|  |  |  |  |  |  |
|  | LPf-LNac | 8.682±0.164 | 8.737±0.215 | 0.278 | 0.040 |
|  |  |  |  |  |  |
|  | LPf-LSTN | 8.577±0.192 | 8.670±0.232 | 0.197 | 0.057 |
|  |  |  |  |  |  |
|  | RPf-RDpu | 8.468±0.253 | 8.607±0.304 | 0.130 | 0.077 |
|  |  |  |  |  |  |
|  | RPf-RNac | 8.662±0.203 | 8.706±0.261 | 0.428 | 0.022 |
|  |  |  |  |  |  |
|  | RPf-RSTN | 8.510±0.212 | 8.636±0.245 | 0.079 | 0.103 |
|  |  |  |  |  |  |
| MD×10^-4^ | LPf-LDpu | 5.544±0.173 | 5.735±0.194 | **0.014** | 0.190 |
|  |  |  |  |  |  |
|  | LPf-LNac | 5.786±0.148 | 5.950±0.207 | **0.010** | 0.210 |
|  |  |  |  |  |  |
|  | LPf-LSTN | 5.429±0.158 | 5.629±0.178 | **0.004** | 0.256 |
|  |  |  |  |  |  |
|  | RPf-RDpu | 5.509±0.169 | 5.695±0.234 | **0.021** | 0.170 |
|  |  |  |  |  |  |
|  | RPf-RNac | 5.826±0.190 | 5.961±0.223 | 0.073 | 0.107 |
|  |  |  |  |  |  |
|  | RPf-RSTN | 5.355±0.163 | 5.583±0.213 | **0.002** | 0.275 |
|  |  |  |  |  |  |
| RD×10^-4^ | LPf-LDpu | 4.038±0.179 | 4.248±0.192 | **0.011** | 0.201 |
|  |  |  |  |  |  |
|  | LPf-LNac | 4.338±0.155 | 4.556±0.231 | **0.003** | 0.261 |
|  |  |  |  |  |  |
|  | LPf-LSTN | 3.855±0.154 | 4.108±0.181 | **<0.001** | 0.339 |
|  |  |  |  |  |  |
|  | RPf-RDpu | 4.030±0.158 | 4.239±0.233 | **0.011** | 0.201 |
|  |  |  |  |  |  |
|  | RPf-RNac | 4.441(0.243) | 4.541(0.169) | **0.035** | 0.144 |
|  |  |  |  |  |  |
|  | RPf-RSTN | 3.777±0.170 | 4.056±0.228 | **<0.001** | 0.318 |
|  |  |  |  |  |  |

Continuous variables are expressed as median (interquartile range-IQR) for non-normally distributed data and as mean ± standard deviation for normally distributed data.

DTI metrics in the Pf pathways are compared between PIGD-FOG and PIGD-nFOG groups using general linear models, controlling for age, disease duration, and MDS-UPDRS Part III. Bold values indicate statistically significant differences between groups (P < 0.05).

**Supplementary Table 2B. NODDI changes** **in PIGD patients with and without FOG**

| NODDI metrics | pathways | PIGD-nFOG(n = 13) | PIGD-FOG(n = 21) | P-value | effect size(partial η^2^) |
| --- | --- | --- | --- | --- | --- |
| NDI×10^-1^ | LPf-LDpu | 6.359(0.570) | 5.892(0.390) | 0.059 | 0.118 |
|  |  |  |  |  |  |
|  | LPf-LNac | 6.029(0.395) | 5.613(0.471) | **0.039** | 0.139 |
|  |  |  |  |  |  |
|  | LPf-LSTN | 6.386±0.390 | 6.076±0.273 | **0.019** | 0.175 |
|  |  |  |  |  |  |
|  | RPf-RDpu | 6.244±0.370 | 5.988±0.333 | 0.057 | 0.119 |
|  |  |  |  |  |  |
|  | RPf-RNac | 5.851±0.345 | 5.693±0.290 | 0.148 | 0.071 |
|  |  |  |  |  |  |
|  | RPf-RSTN | 6.505±0.393 | 6.185±0.308 | **0.019** | 0.175 |
|  |  |  |  |  |  |
| ODI×10^-1^ | LPf-LDpu | 2.670±0.121 | 2.683±0.123 | 0.874 | 0.001 |
|  |  |  |  |  |  |
|  | LPf-LNac | 2.846±0.147 | 2.902±0.129 | 0.270 | 0.042 |
|  |  |  |  |  |  |
|  | LPf-LSTN | 2.578±0.099 | 2.674±0.137 | 0.163 | 0.066 |
|  |  |  |  |  |  |
|  | RPf-RDpu | 2.711±0.143 | 2.757±0.180 | 0.811 | 0.002 |
|  |  |  |  |  |  |
|  | RPf-RNac | 2.929±0.158 | 3.009±0.231 | 0.517 | 0.015 |
|  |  |  |  |  |  |
|  | RPf-RSTN | 2.609±0.120 | 2.662±0.176 | 0.668 | 0.006 |
|  |  |  |  |  |  |
| FWF×10^-1^ | LPf-LDpu | 3.397±0.289 | 3.348±0.293 | 0.620 | 0.009 |
|  |  |  |  |  |  |
|  | LPf-LNac | 3.598(0.300) | 3.627(0.482) | 0.885 | <0.001 |
|  |  |  |  |  |  |
|  | LPf-LSTN | 3.335(0.233) | 3.387(00.391) | 0.938 | <0.001 |
|  |  |  |  |  |  |
|  | RPf-RDpu | 3.460±0.211 | 3.483±0.223 | 0.907 | <0.001 |
|  |  |  |  |  |  |
|  | RPf-RNac | 3.749±0.180 | 3.821±0.299 | 0.773 | 0.003 |
|  |  |  |  |  |  |
|  | RPf-RSTN | 3.482±0.163 | 3.489±0.233 | 0.789 | 0.003 |
|  |  |  |  |  |  |

Continuous variables are expressed as median (interquartile range-IQR) for non-normally distributed data and as mean ± standard deviation for normally distributed data.

NODDI metrics in the Pf pathways are compared between PIGD-FOG and PIGD-nFOG groups using general linear models, controlling for age, disease duration, and MDS-UPDRS Part III. Bold values indicate statistically significant differences between groups (P < 0.05).

**Supplementary Table 3A. DTI changes in PIGD patients with and without FOG** **after additional adjustment for motor symptom laterality**

| DTI metrics | pathways | P-value | effect size(partial η^2^) |
| --- | --- | --- | --- |
| FA×10^-1^ | LPf-LDpu | **0.020** | 0.179 |
|  | LPf-LNac | **0.013** | 0.201 |
|  | LPf-LSTN | **0.002** | 0.300 |
|  | RPf-RDpu | **0.006** | 0.243 |
|  | RPf-RNac | **0.004** | 0.255 |
|  | RPf-RSTN | **<0.001** | 0.372 |
| AD×10^-4^ | LPf-LDpu | **0.044** | 0.137 |
|  | LPf-LNac | 0.225 | 0.052 |
|  | LPf-LSTN | 0.172 | 0.066 |
|  | RPf-RDpu | 0.104 | 0.092 |
|  | RPf-RNac | 0.381 | 0.027 |
|  | RPf-RSTN | **0.042** | 0.140 |
| MD×10^-4^ | LPf-LDpu | **0.009** | 0.219 |
|  | LPf-LNac | **0.008** | 0.227 |
|  | LPf-LSTN | **0.003** | 0.274 |
|  | RPf-RDpu | **0.022** | 0.175 |
|  | RPf-RNac | 0.073 | 0.111 |
|  | RPf-RSTN | **0.002** | 0.290 |
| RD×10^-4^ | LPf-LDpu | **0.008** | 0.223 |
|  | LPf-LNac | **0.003** | 0.270 |
|  | LPf-LSTN | **<0.001** | 0.353 |
|  | RPf-RDpu | **0.013** | 0.200 |
|  | RPf-RNac | **0.038** | 0.144 |
|  | RPf-RSTN | **0.001** | 0.319 |

DTI metrics in the Pf pathways are compared between PIGD-FOG and PIGD-nFOG groups using general linear models, controlling for age, disease duration, MDS-UPDRS Part III, and motor symptom laterality. Bold values indicate statistically significant differences between groups (P < 0.05).

Descriptive statistics for these variables are provided in Supplementary Table 2A.

**Supplementary Table 3B. NODDI changes in PIGD patients with and without FOG** **after additional adjustment for motor symptom laterality**

| NODDI metrics | pathways | P-value | effect size(partial η^2^) |
| --- | --- | --- | --- |
| NDI×10^-1^ | LPf-LDpu | **0.043** | 0.138 |
|  | LPf-LNac | **0.033** | 0.152 |
|  | LPf-LSTN | **0.015** | 0.194 |
|  | RPf-RDpu | 0.056 | 0.124 |
|  | RPf-RNac | 0.146 | 0.074 |
|  | RPf-RSTN | **0.018** | 0.184 |
| ODI×10^-1^ | LPf-LDpu | 0.844 | 0.001 |
|  | LPf-LNac | 0.294 | 0.039 |
|  | LPf-LSTN | 0.178 | 0.064 |
|  | RPf-RDpu | 0.866 | 0.001 |
|  | RPf-RNac | 0.550 | 0.013 |
|  | RPf-RSTN | 0.706 | 0.005 |
| FWF×10^-1^ | LPf-LDpu | 0.559 | 0.012 |
|  | LPf-LNac | 0.957 | <0.001 |
|  | LPf-LSTN | 0.997 | <0.001 |
|  | RPf-RDpu | 0.873 | <0.001 |
|  | RPf-RNac | 0.806 | 0.002 |
|  | RPf-RSTN | 0.761 | 0.003 |

NODDI metrics in the Pf pathways are compared between PIGD-FOG and PIGD-nFOG groups using general linear models, controlling for age, disease duration, MDS-UPDRS Part III, and motor symptom laterality. Bold values indicate statistically significant differences between groups (P < 0.05).

Descriptive statistics for these variables are provided in Supplementary Table 2B.

**Supplementary Table 4A. DTI changes between PIGD-nFOG and PDTD patients**

| DTI metrics | pathways | PIGD-nFOG(n = 13) | PDTD(n = 25) | P-value | effect size(partial η^2^) |
| --- | --- | --- | --- | --- | --- |
| FA×10^-1^ | LPf-LDpu | 4.777±0.210 | 4.681±0.153 | **0.036** | 0.126 |
|  |  |  |  |  |  |
|  | LPf-LNac | 4.404±0.223 | 4.303±0.169 | **0.046** | 0.115 |
|  |  |  |  |  |  |
|  | LPf-LSTN | 4.900(0.324) | 4.905(0.198) | 0.127 | 0.069 |
|  |  |  |  |  |  |
|  | RPf-RDpu | 4.730±0.170 | 4.649±0.168 | **0.042** | 0.119 |
|  |  |  |  |  |  |
|  | RPf-RNac | 4.237(0.230) | 4.285(0.271) | 0.108 | 0.076 |
|  |  |  |  |  |  |
|  | RPf-RSTN | 4.960±0.156 | 4.906±0.180 | 0.142 | 0.064 |
|  |  |  |  |  |  |
| AD×10^-4^ | LPf-LDpu | 8.556±0.194 | 8.502±0.227 | 0.641 | 0.007 |
|  |  |  |  |  |  |
|  | LPf-LNac | 8.682±0.164 | 8.619±0.195 | 0.453 | 0.017 |
|  |  |  |  |  |  |
|  | LPf-LSTN | 8.577±0.192 | 8.531±0.248 | 0.794 | 0.002 |
|  |  |  |  |  |  |
|  | RPf-RDpu | 8.468±0.253 | 8.424±0.235 | 0.847 | 0.001 |
|  |  |  |  |  |  |
|  | RPf-RNac | 8.662±0.203 | 8.599±0.232 | 0.721 | 0.004 |
|  |  |  |  |  |  |
|  | RPf-RSTN | 8.510±0.212 | 8.508±0.226 | 0.843 | 0.001 |
|  |  |  |  |  |  |
| MD×10^-4^ | LPf-LDpu | 5.544±0.173 | 5.562±0.183 | 0.476 | 0.016 |
|  |  |  |  |  |  |
|  | LPf-LNac | 5.786±0.148 | 5.814±0.210 | 0.377 | 0.024 |
|  |  |  |  |  |  |
|  | LPf-LSTN | 5.429±0.158 | 5.463±0.201 | 0.299 | 0.033 |
|  |  |  |  |  |  |
|  | RPf-RDpu | 5.523(0.328) | 5.462(0.192) | 0.377 | 0.024 |
|  |  |  |  |  |  |
|  | RPf-RNac | 5.845(0.287) | 5.810(0.228) | 0.346 | 0.027 |
|  |  |  |  |  |  |
|  | RPf-RSTN | 5.373(0.247) | 5.395(0.206) | 0.168 | 0.057 |
|  |  |  |  |  |  |
| RD×10^-4^ | LPf-LDpu | 4.014(0.285) | 4.070(0.275) | 0.231 | 0.043 |
|  |  |  |  |  |  |
|  | LPf-LNac | 4.340(0.172) | 4.370(0.298) | 0.145 | 0.063 |
|  |  |  |  |  |  |
|  | LPf-LSTN | 3.886(0.228) | 3.891(0.246) | 0.128 | 0.069 |
|  |  |  |  |  |  |
|  | RPf-RDpu | 4.008(0.200) | 4.022(0.174) | 0.149 | 0.062 |
|  |  |  |  |  |  |
|  | RPf-RNac | 4.441(0.243) | 4.388(0.238) | 0.156 | 0.060 |
|  |  |  |  |  |  |
|  | RPf-RSTN | 3.836(0.215) | 3.846(0.223) | 0.079 | 0.090 |
|  |  |  |  |  |  |

Continuous variables are expressed as median (interquartile range-IQR) for non-normally distributed data and as mean ± standard deviation for normally distributed data.

DTI metrics in the Pf pathways are compared between PIGD-nFOG and PDTD groups using general linear models, controlling for age, disease duration, and MDS-UPDRS Part III. Bold values indicate statistically significant differences between groups (P < 0.05).

**Supplementary Table 4B. NODDI changes between PIGD-nFOG and PDTD patients**

| NODDI metrics | pathways | PIGD-nFOG(n = 13) | PDTD(n = 25) | P-value | effect size(partial η^2^) |
| --- | --- | --- | --- | --- | --- |
| NDI×10^-1^ | LPf-LDpu | 6.359(0.570) | 6.161(0.443) | 0.641 | 0.007 |
|  |  |  |  |  |  |
|  | LPf-LNac | 6.029(0.395) | 5.859(0.472) | 0.611 | 0.008 |
|  |  |  |  |  |  |
|  | LPf-LSTN | 6.386±0.390 | 6.346±0.348 | 0.474 | 0.016 |
|  |  |  |  |  |  |
|  | RPf-RDpu | 6.318(0.551) | 6.301(0.472) | 0.649 | 0.006 |
|  |  |  |  |  |  |
|  | RPf-RNac | 5.916(0.436) | 5.901(0.373) | 0.545 | 0.011 |
|  |  |  |  |  |  |
|  | RPf-RSTN | 6.583(0.550) | 6.484(0.346) | 0.425 | 0.019 |
|  |  |  |  |  |  |
| ODI×10^-1^ | LPf-LDpu | 2.650(0.243) | 2.700(0.124) | 0.336 | 0.028 |
|  |  |  |  |  |  |
|  | LPf-LNac | 2.780(0.247) | 2.824(0.206) | 0.374 | 0.024 |
|  |  |  |  |  |  |
|  | LPf-LSTN | 2.591(0.130) | 2.583(0.154) | 0.273 | 0.036 |
|  |  |  |  |  |  |
|  | RPf-RDpu | 2.687(0.205) | 2.725(0.214) | 0.170 | 0.056 |
|  |  |  |  |  |  |
|  | RPf-RNac | 2.981(0.230) | 2.895(0.211) | 0.435 | 0.019 |
|  |  |  |  |  |  |
|  | RPf-RSTN | 2.587(0.168) | 2.591(0.195) | 0.568 | 0.010 |
|  |  |  |  |  |  |
| FWF×10^-1^ | LPf-LDpu | 3.347(0.253) | 3.323(0.324) | 0.774 | 0.003 |
|  |  |  |  |  |  |
|  | LPf-LNac | 3.598(0.300) | 3.575(0.343) | 0.521 | 0.013 |
|  |  |  |  |  |  |
|  | LPf-LSTN | 3.335(0.233) | 3.387(0.243) | 0.665 | 0.006 |
|  |  |  |  |  |  |
|  | RPf-RDpu | 3.496(0.264) | 3.424(0.280) | 0.616 | 0.008 |
|  |  |  |  |  |  |
|  | RPf-RNac | 3.756(0.272) | 3.655(0.401) | 0.869 | <0.001 |
|  |  |  |  |  |  |
|  | RPf-RSTN | 3.506(0.272) | 3.375(0.240) | 0.952 | <0.001 |
|  |  |  |  |  |  |

Continuous variables are expressed as median (interquartile range-IQR) for non-normally distributed data and as mean ± standard deviation for normally distributed data.

NODDI metrics in the Pf pathways are compared between PIGD-nFOG and PDTD groups using general linear models, controlling for age, disease duration, and MDS-UPDRS Part III. Bold values indicate statistically significant differences between groups (P < 0.05).

**Supplementary Table 5.** **ROC analyses of different models for discriminating PIGD-FOG from PIGD-nFOG**

| Variable | AUC | 95%CI | Sensitivity,% | Specificity,% |  |
| --- | --- | --- | --- | --- | --- |
| Pf-Dpu | 0.857 | 0.727-0.987 | 76.2 | 84.6 |  |
|  |  |  |  |  |  |
| Pf-Nac | 0.850 | 0.722-0.978 | 66.7 | 100.0 |  |
|  |  |  |  |  |  |
| Pf-STN | 0.927 | 0.844-1.000 | 81.0 | 92.3 |  |
|  |  |  |  |  |  |

Separate ROC curves are generated for each Pf pathway (Pf-Dpu, Pf-Nac, Pf-STN) by combining microstructural metrics showing significant intergroup differences in either hemisphere within that pathway.

**Supplementary Table 6. Partial correlations between imaging metrics and MDS-UPDRS Part III scores in the PIGD subtype after additional adjustment for LEDD**

| metrics | pathways | P-value | r |  |
| --- | --- | --- | --- | --- |
|  |  |  |  |  |
| FA | LPf-LSTN | 0.058 | -0.350 |  |
|  | RPf-RDpu | 0.204 | -0.239 |  |
|  | RPf-RSTN | 0.071 | -0.329 |  |
| AD | LPf-LDpu | 0.721 | 0.068 |  |
|  | LPf-LNac | 0.444 | -0.145 |  |
|  | LPf-LSTN | 0.903 | -0.023 |  |
|  | RPf-RSTN | 0.870 | 0.031 |  |
| MD | LPf-LDpu | 0.392 | 0.162 |  |
|  | LPf-LSTN | 0.571 | 0.108 |  |
|  | RPf-RSTN | 0.289 | 0.200 |  |
| RD | LPf-LSTN | 0.444 | 0.145 |  |
| NDI | LPf-LDpu | 0.257 | -0.214 |  |
|  | LPf-LSTN | 0.311 | -0.192 |  |
|  | RPf-RDpu | 0.216 | -0.233 |  |
|  | RPf-RSTN | 0.276 | -0.206 |  |

Partial correlation analyses are performed between imaging metrics and MDS-UPDRS Part III scores in the PIGD subtype, controlling for age, sex, years of education, and LEDD.

**Supplementary Table 7. ROC analyses of different models for discriminating PIGD-FOG from HC**

| Variable | AUC | 95%CI | Sensitivity,% | Specificity,% |  |
| --- | --- | --- | --- | --- | --- |
| Pf-Dpu | 0.914 | 0.840-0.987 | 90.5 | 78.9 |  |
|  |  |  |  |  |  |
| Pf-Nac | 0.915 | 0.843-0.987 | 76.2 | 97.4 |  |
|  |  |  |  |  |  |
| Pf-STN | 0.959 | 0.916-1.000 | 95.2 | 84.2 |  |
|  |  |  |  |  |  |

Separate ROC curves are generated for each Pf pathway (Pf-Dpu, Pf-Nac, Pf-STN) by combining the microstructural metrics that showed significant differences between PIGD-FOG and PIGD-nFOG in either hemisphere within the same pathway. These selected metrics are then used to construct classification models for distinguishing PIGD-FOG patients from HC.


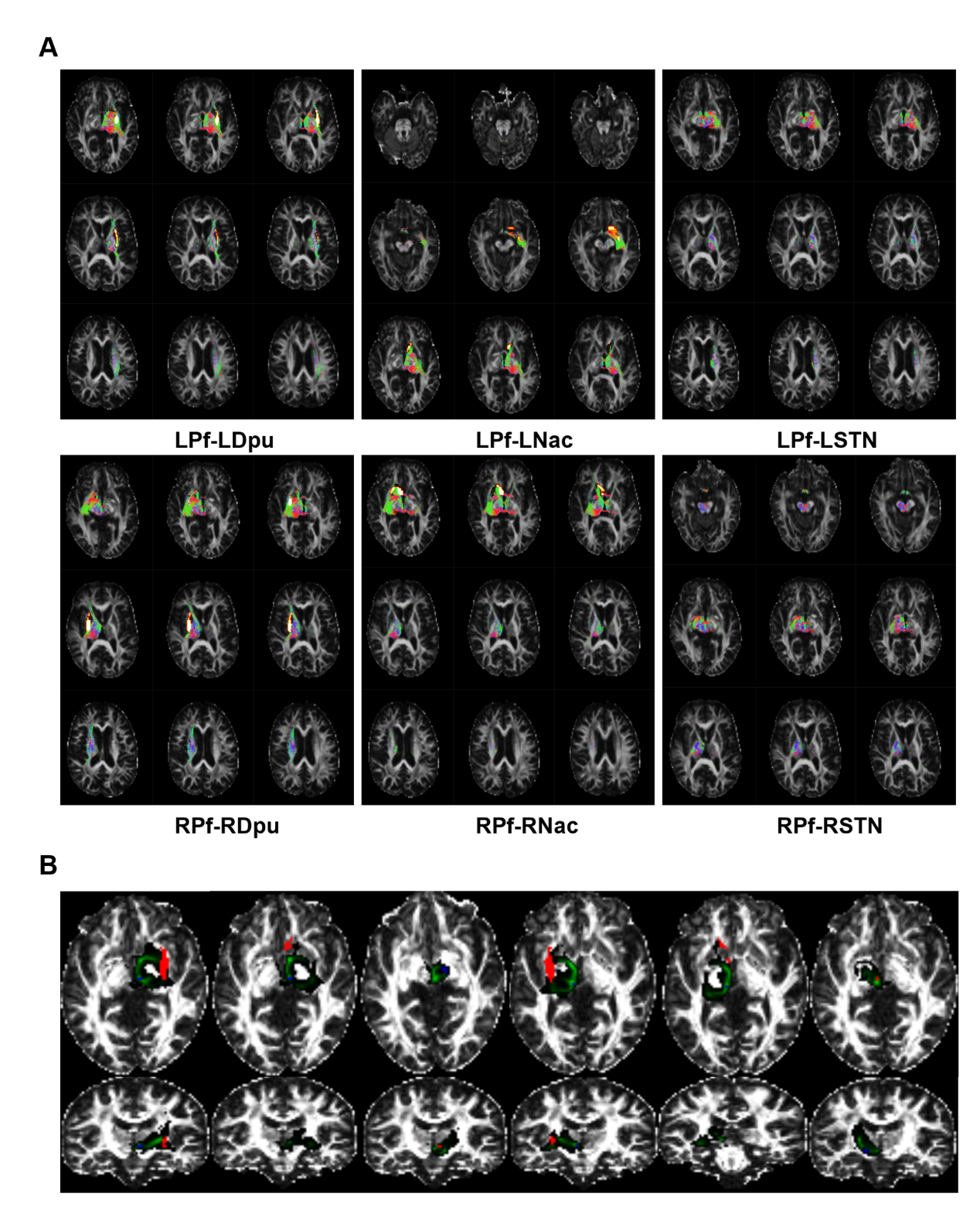


**Supplementary Fig. 1 Fiber tractography results**

Supplementary Fig. 1A. Fiber tractography results are overlaid on individual-space FA images. Fiber pathways are displayed in color, representing the reconstructed tracts. Regions of interest (ROIs) are highlighted in bright orange.

Supplementary Fig. 1B. The fiber tract maps are registered to the voxel space, with red and blue indicating the corresponding ROIs. The upper panel shows axial views, and the lower panel shows the corresponding coronal views. From left to right, the fiber tractography results correspond to LPf-LDpu, LPf-LNac, LPf-LSTN, RPf-RDpu, RPf-RNac, and RPf-RSTN.

LPf, left parafascicular nucleus; LDpu, left dorsal putamen; LNac, left nucleus accumbens; LSTN, left subthalamic nucleus; RPf, right parafascicular nucleus; RDpu, right dorsal putamen; RNac, right nucleus accumbens; RSTN, right subthalamic nucleus; L, left; R, right.


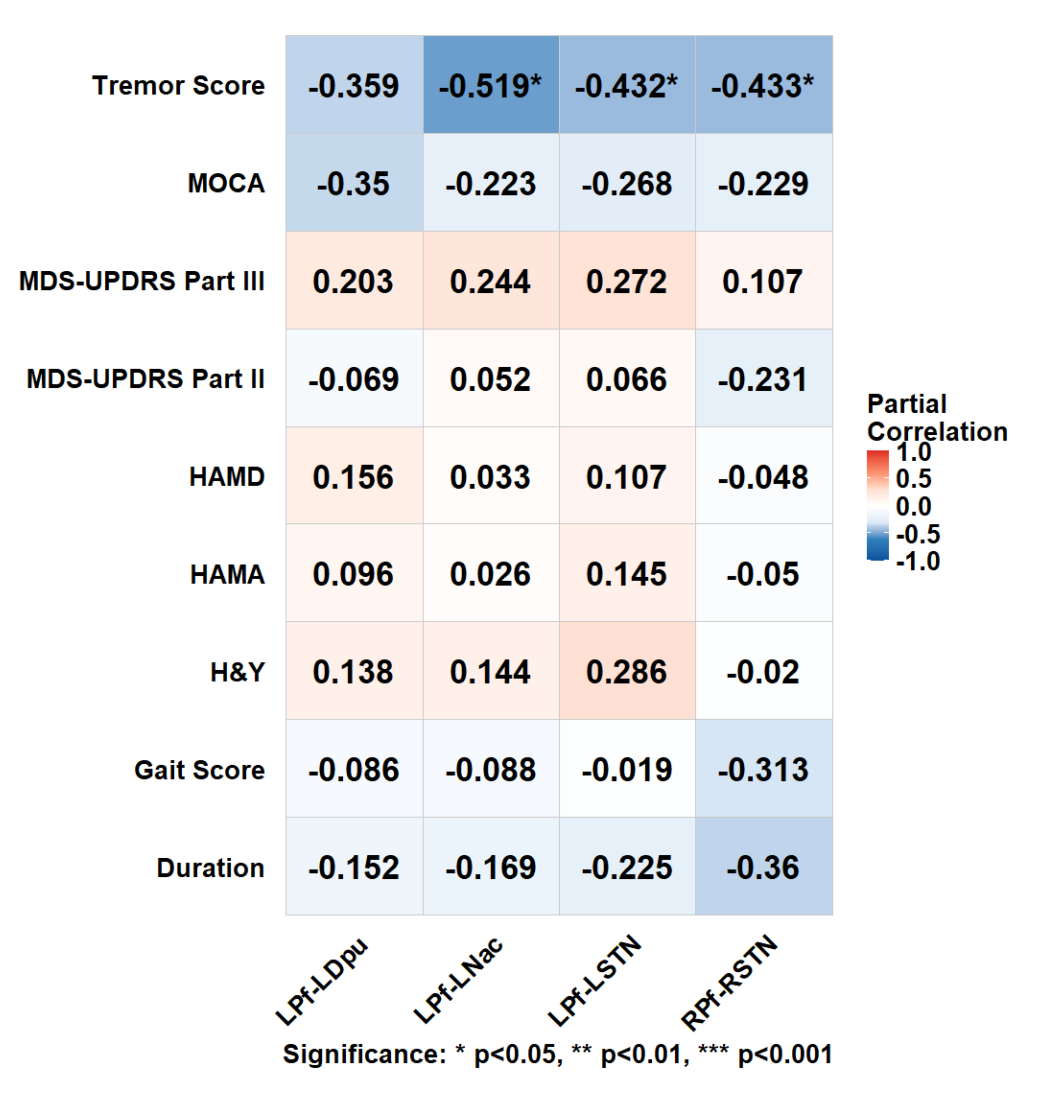


**Supplementary Fig. 2** **Partial correlations between imaging metrics and clinical measures in the PDTD** **subtype (n = 25)**

LPf, left parafascicular nucleus; LDpu, left dorsal putamen; LNac, left nucleus accumbens; LSTN, left subthalamic nucleus; RPf, right parafascicular nucleus; RDpu, right dorsal putamen; RNac, right nucleus accumbens; RSTN, right subthalamic nucleus; L, left; R, right; H&Y, Hoehn and Yahr disability scale; MDS-UPDRS Part II, Movement Disorder Society Unified Parkinson’s Disease Rating Scale, part II; MDS-UPDRS Part III, Movement Disorder Society Unified Parkinson’s Disease Rating Scale, part III; MOCA, Montreal Cognitive Assessment; HAMD, Hamilton Depression Scale; HAMA, Hamilton Anxiety Scale.

Significance: *, P <0.05, **, P <0.01; ***, P <0.001.


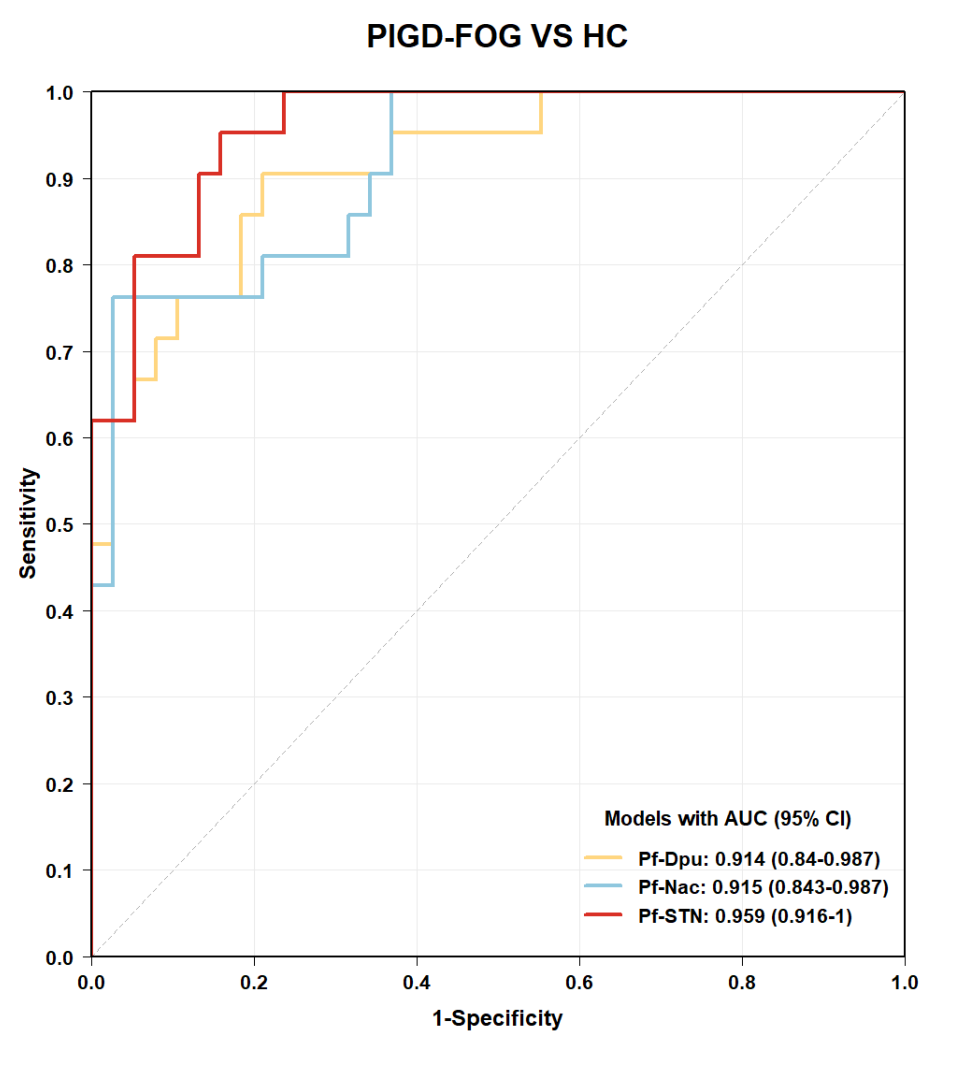


**Supplementary Fig. 3 Discriminative performance of multiple models for distinguishing PIGD-FOG (n = 21) from HC (n = 38) using ROC curve analyses.**

Separate ROC curves are generated for each Pf pathway (Pf-Dpu, Pf-Nac, Pf-STN) by combining the microstructural metrics that showed significant differences between PIGD-FOG and PIGD-nFOG (n = 13) in either hemisphere within the same pathway. These selected metrics are then used to construct classification models for distinguishing PIGD-FOG patients from HC.

**Reference**

[1] Su W, Li K, Li C-M, et al. Motor Symptom Lateralization Influences Cortico-Striatal Functional Connectivity in Parkinson’s Disease[J]. Frontiers in Neurology, 2021, 12: 619631.
